# Mapping symptom-general and symptom-specific targets for transcranial magnetic stimulation in schizophrenia: an electric-field modeling meta-analysis

**DOI:** 10.1038/s41380-025-03238-z

**Published:** 2025-09-22

**Authors:** Lorina Sinanaj, Konstantinos Pallis, Anahita Fazel Dehkordi, Philippe Huguelet, Stefan Kaiser, Indrit Bègue

**Affiliations:** 1 Laboratory for Neuroimaging and Translational Psychiatry, Department of Psychiatry, University of Geneva, Geneva, Switzerland; 2 Synapsy Center for Neuroscience and Mental Health Research, University of Geneva, Geneva, Switzerland; 3 Adult Psychiatry Division, Department of Psychiatry, University Hospitals of Geneva, Geneva, Switzerland; 4 Laboratory for Experimental Psychopathology, Department of Psychiatry, University of Geneva, Geneva, Switzerland

**Keywords:** Schizophrenia, Neuroscience

## Abstract

Negative, positive, and cognitive symptoms of schizophrenia relate to disruptions in partially distinct brain circuits. Although promising, transcranial magnetic stimulation (TMS) strategies across and within symptom domains remain to be established due to TMS protocol heterogeneity. For this, we combined standard meta-analysis with electric field (E-field) modeling to identify stimulation sites where E-field strength was associated most significantly with clinical improvement. Standard meta-analysis of randomized, sham-controlled studies in 4283 patients demonstrated the benefit of TMS across symptom domains, regardless of target or protocol. TMS significantly improved negative and cognitive symptoms with high-frequency stimulation applied to the left prefrontal cortex, whereas positive symptoms improved with low-frequency TMS applied to the left temporoparietal cortex. In-depth examination of these results with E-field modeling identified stimulation of the left dorsomedial prefrontal cortex (L-DMPFC), left orbitofrontal cortex (L-OFC), and left cerebellar crus II and right lobule IX to be significantly associated with improvement across all symptom domains. Greater overlap of studies’ stimulation targets with L-DMPFC and L-OFC related to improved outcomes. For negative symptoms, E-field distribution in L-DMPFC and L-OFC related most significantly to clinical improvement. Greater proximity to L-DMPFC stimulation site indicated better outcomes, with trend-level significance for L-OFC. In the cognitive domain, E-field distribution in the left dorsolateral prefrontal cortex was related to clinical improvement. Finally, the strongest E-field association with clinical improvement was found in the right cerebellar lobules VIIIA, VIIIB, and IX for positive symptoms. These results support symptom-general and symptom-specific TMS approaches for distinct therapeutic goals towards personalized neuromodulation in schizophrenia.

## Introduction

Schizophrenia is a chronic and debilitating disorder affecting up to 1% of the general population. It presents with diverse clinical manifestations including hallucinations and delusions (positive symptoms), apathy and diminished verbal and emotional expression (negative symptoms), and cognitive deficits affecting working memory, attention and processing speed. Schizophrenia has the highest individual burden of all psychiatric disorders [1], imposing significant economic costs [2] and reducing life expectancy by up to 15 years [3]. Notably, cognitive and negative symptoms are associated with worse psychosocial functioning [4] highlighting the urgent need for more effective treatments.

While antipsychotic medications primarily target positive symptoms, they show limited efficacy for negative and cognitive symptoms and may even worsen these deficits [5, 6], emphasizing the need for alternative approaches like transcranial magnetic stimulation (TMS). TMS modulates neural activity through electromagnetic pulses, and has shown promise in targeting symptom-specific neural circuits [7, 8]. The heterogeneity of symptom presentation in schizophrenia complicates therapeutic decisions, as current approaches generally lack a framework that addresses specific symptom dimensions. This gap highlights the need to differentiate between symptom-general and symptom-specific targets within TMS therapy. Symptom-general targets could be suited for patients with mixed clinical presentation, while symptom-specific targets could allow for precise interventions focused on particularly distressing or disabling symptoms. For instance, TMS targeting auditory processing circuits could help a patient with persistent hallucinations, whereas stimulation aimed at motivational circuits may benefit someone with severe avolition. However, many patients present with mixed symptom profiles, where both specific and broad symptom domains overlap. Such distinction raises an opportunity to tailor TMS to individual symptom profiles, potentially enabling a more personalized and effective approach.

Current TMS studies often rely on standard anatomical or scalp-based targeting, which may fail to account for individual neural circuitry underlying each symptom domain [9, 10]. Therefore, TMS protocols have been applied to varied sites such as primary auditory cortices (e.g., left superior temporal gyrus; STG) to counter auditory hallucinations [11], to distinct nodes in the cerebellar-prefrontal circuit for negative symptoms [12] and to dorsolateral prefrontal cortex for cognitive symptoms [13] with heterogeneous outcomes [14]. Evidence from depression studies indicates that aligning TMS targets with neuroimaging-defined locations enhances treatment outcomes, underscoring the potential benefits of individualized TMS targeting [15, 16]. Nevertheless, a comprehensive examination of TMS stimulation targets across and within all symptom domains in schizophrenia is still lacking and could be essential for identifying optimal brain sites for diverse therapeutic goals with TMS.

Beyond spatial targeting considerations, another source of the variability in TMS response can result from differences in stimulation parameters, such as coil type, position, and intensity, all of which shape the electric field (E-field) distribution and therapeutic effect. Optimizing these parameters has shown enhanced outcomes in major depressive disorder and auditory verbal hallucinations in schizophrenia [17]. Advances in meta-analytic methods using E-field strength from transcranial direct current stimulation (tDCS) have mapped E-field distributions across the brain and linked these to behavioral outcomes (e.g., working memory) [18, 19] and clinical improvements [20], by identifying neural areas with the highest tDCS-induced effects. However, no study has yet examined whether TMS variability in schizophrenia could be optimized through integrating E-field modeling with meta-analytic data to identify neural areas linked to improvement in schizophrenia symptom domains.

To address these challenges, we capitalized on an established combined method that integrates E-field modeling with a standard meta-analytic approach to identify optimal TMS sites in schizophrenia. Specifically, we first conducted a *standard* meta-analysis to assess TMS efficacy across and within distinct symptoms of schizophrenia and explored potential factors of heterogeneity such as stimulation target and protocol frequency with subgroup analyses. We then simulated E-field distributions and identified brain sites where TMS-induced E-fields most strongly correlated with symptom improvement in schizophrenia. We hypothesized that symptom-specific neural circuits would be associated with TMS therapeutic efficacy in schizophrenia. Specifically, we expected that stimulation of prefrontal regions and the cerebellum would yield significant improvements in negative and cognitive symptoms, whereas targeting the auditory cortices would be linked to superior outcomes for positive symptoms, such as auditory hallucinations. Furthermore, we proposed that TMS studies with targeting locations closer to the E‑field modeling-derived optimal site would demonstrate greater clinical improvement, thereby validating our approach.

## Materials and methods

### Eligibility criteria and search strategy

**Fig. 1:**
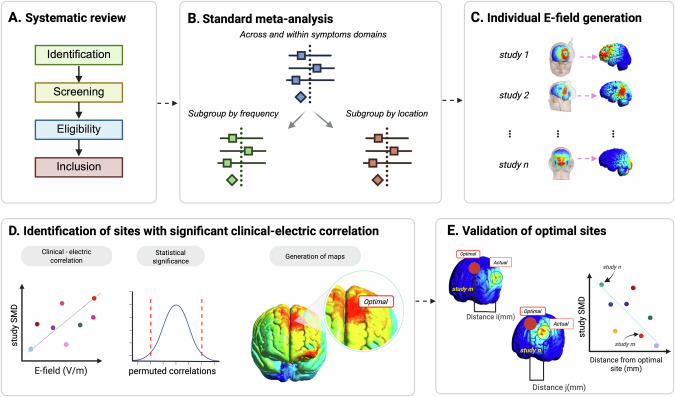
Rationale for optimal TMS targeting in schizophrenia based on E-field modeling. **A** Systematic review of randomized, sham-controlled trials. **B** Standard meta-analysis across and within symptom domains, followed by subgroup analysis focused on TMS location and frequency. **C** Simulation of E-fields for each study, according to specific TMS parameters given in the study. **D** For each brain tetrahedral element, the correlation between clinical effect size and E-field strength (CEC) is measured. Following the calculation of CEC values, a 2-sided permutation test is employed to statistically assess their significance. All CEC values are displayed on the gray matter volume to identify optimal brain sites where TMS has the most beneficial impact. **E** Closer distance between actual targets and optimal TMS site is associated with improved clinical effect. Created with BioRender.com.

We aimed to identify optimal non-invasive stimulation sites for treatment across and within the negative, cognitive, and positive symptom domains of schizophrenia. First, we conducted a systematic review following PRISMA protocols (Preferred Reporting Items for Systematic Reviews and Meta-Analyses) (illustrated in Fig. 1A). We screened the literature with the following eligibility criteria. We included randomized, sham-controlled trials of TMS in adults (≥18 years) diagnosed with a schizophrenia spectrum disorder (e.g., schizophrenia, schizoaffective disorder, or psychosis). We only included studies for which the outcome (i.e., change in negative, positive or cognitive domains) was the *primary* endpoint. In other words, we did not include a study in one domain (e.g., negative) if the study was aimed to study another domain (e.g., positive). Focusing on primary endpoints minimizes confounding factors and follows strict meta-analytic standards. We excluded studies where TMS was co-initiated at the same time with another therapeutic intervention. Regarding outcome measurements, we used established instruments to assess negative, cognitive, and positive symptoms; a comprehensive list of these measures is provided in Appendix S1. The systematic review is registered on PROSPERO (https://www.crd.york.ac.uk/prospero/display_record.php?RecordID=540178).

We systematically searched PubMed, EMBASE, PsycINFO, and Web of Science up to March 5, 2025, using schizophrenia and TMS-related keywords (complete search strings are in Appendix S1). Titles and abstracts were screened independently by two authors (KP and IB), followed by full-text evaluation of potentially relevant studies. KP and AFD extracted key study details, including demographics, TMS parameters, outcome measures, and follow-up scores. For studies using scalp-based targeting, MNI coordinates were derived from the OPT-TMS study [21]. Missing data were requested via email by KP or digitally extracted using GetData Graph Digitizer (http://getdata-graph-digitizer.com/). Studies lacking necessary post-treatment outcome data for effect size calculation were excluded. A detailed PRISMA flow diagram summarizing the study selection process, from 4311 initially identified articles to the final inclusion of 107 studies, is available in the Supplementary Material (Figure S1). Summary tables of all the studies for each symptom domain can be found in the Supplementary Material. Study bias was evaluated using the Cochrane Risk of Bias Tool 2.0 (https://methods.cochrane.org/risk-bias-2); complete details are provided in Table S4. Further details of the search strategy, data extraction process and risk of bias assessment are provided in Appendix S1.

### Effect size extraction and standard meta-analysis

We evaluated the effect size of TMS by analyzing changes in symptom severity across negative, cognitive, and positive domains of the active arm compared to a sham control (Fig. 1B). To accurately capture treatment effects, we analyzed the mean changes in symptom severity scores rather than simply the final endpoint score which may be confounded for baseline symptom severity for both treatment and sham groups. Studies lacking baseline or endpoint scores were omitted from our analysis (see Appendix S2). For consistency of interpretation, in our meta-analysis a positive mean change across all domains indicates improvement. Thus, for negative and positive symptoms, where a decrease in clinical scores indicates improvement, we calculated the mean change as the initial score (baseline) minus the final score (endpoint). For cognitive symptoms, where an increase in scores is better, we calculated mean change as the final score minus the initial score.

To quantify the effect size, we used the standardized mean difference (SMD), calculated as Cohen’s d, which normalizes the difference between the treatment and sham mean changes by the pooled standard deviation (SD):

$${SMD}=\frac{{Mea}{n}_{{treatment}}-{Mea}{n}_{{sham}}}{S{D}_{{pooled}}}$$

For studies with small sample sizes (total participants <50, specifically 61.8% of all the included studies in our meta-analysis), we applied Hedges’ correction factor [22] to adjust for bias in effect size estimation. A breakdown of sample sizes across all the included studies can be found in Table S5. We assigned a single SMD to each study to represent the effect size associated with each TMS montage. A positive SMD indicates that TMS has effectively improved symptom severity, while an SMD of zero or negative suggests no improvement.

To address the high heterogeneity in TMS studies resulting from variations in participant characteristics, coil specifications and treatment protocols, we employed a random-effects model [23]. This model considers both within-study and between-study variance (tau-squared τ²) to calculate the cumulative effect size SMD and 95% confidence intervals (CI). We assessed the significance of this effect size using the corresponding 95% CI and p-value. Heterogeneity was measured using Cochran’s Q and the I-squared (I²) statistics, adjusted for τ². We then applied a bootstrapping sensitivity analysis to assess the robustness of the pooled SMD in the presence of outliers, and a standard nonparametric permutation test to evaluate its significance under the null hypothesis of no effect. Full details of these procedures are provided in Appendix S3. We also used Kendall’s rank correlation test to evaluate potential publication bias via funnel plot asymmetry test, by examining standard error versus effect size for each symptom domain.

Finally, we performed subgroup analyses focused on location of TMS application and frequency of TMS protocol following previous studies [24].

### Finite Element Modeling (FEM) simulation

We simulated E-field distributions to quantify how different TMS parameters, namely coil type, position, orientation and stimulation intensity, affected targeted brain regions for each study (Fig. 1C). Finite element method (FEM) simulations were conducted using SimNIBS version 4.5 [25] and Python 3.12 to model the E-fields induced by TMS. The MNI152 standard brain template was used to generate E-field simulations. This template is derived from 3D brain MRI images of 152 adult brains, including 86 male and 66 female brains with an average age = 25.02 ± 4.90 and age range = 18–44 years old [26]. The MNI152 template has been recently validated for correctly estimating group-level TMS-induced E-fields [27]. This template provides full head coverage including the cerebellum, a region excluded in previous E-field modeling studies. Bilateral TMS studies were simulated by applying the principle of superposition to the E-fields from the left and right hemispheres [28].

FEM analyses focused on E-field distributions at targets linked to clinical improvement in the standard meta-analysis. For studies using the EEG 10/20 system, the coordinates were transformed to the standard 10/10 electrode positions in SimNIBS. We did not perform FEM simulations for studies using coils or stimulators unsupported in SimNIBS (e.g., Cadwell, YRDCCY-I, Neurosoft Ltd.) or for those lacking essential details (such as coil type, targeting method, inferred coordinates or stimulation intensity); see Appendix S4 for a full list of excluded studies.

### Meta-analytic correlation between E-field distribution and effect size

Expanding on established methodology [18], we analyzed the relationship between E-field strength and SMD at each brain tetrahedral element, quantifying this through the Clinical E-Field Correlation (CEC) (Fig. 1D). This correlation coefficient serves as an indicator of how TMS-induced E-fields influence clinical changes (i.e., improvement) in schizophrenia. Specifically, positive CEC values suggest that higher E-fields in certain brain regions are associated with symptom improvement. Conversely, negative CEC values indicate that lower E-fields in certain regions correlate with symptom improvement, implying that stronger E-field doses in these areas may not be beneficial. CEC values close to zero suggest no significant relationship between E-field strength and symptom improvement in those regions. For correlation analyses, we used the nonparametric Spearman’s rank correlation test as it is less sensitive to outliers and doesn’t make any assumptions about the data distribution.

We calculated CEC values and conducted a two-sided permutation test (p < 0.05) by randomly permuting CEC values across each tetrahedral element 1000 times to create a null distribution. Comparing actual CEC values to this distribution generated p-values for each element. Heatmaps were then produced to visually represent CEC values, highlighting brain sites consistently linked to improved outcomes across studies. Figures are created with BioRender.com [29].

### Validation of E-field modeling derived TMS targets

We assessed the validity of the E-field-modeling–derived TMS sites by examining the overlap between these sites and the actual TMS study targets [15]: studies whose actual TMS targets were closer to the optimal site derived from our E-field modeling approach, would be associated with better treatment outcomes than those further away. To this end, we standardized the location of all clinical stimulation targets (i.e., specified using scalp-based measurements, anatomical landmarks, EEG coordinates or Talairach in the original studies) by mapping them to MNI coordinates in mm [x, y, z] for each study. Next, we computed the Euclidean distance between individual actual TMS targets and the optimal site and correlated this distance with the respective study’s standardized mean difference in clinical outcomes (Fig. 1E). Associations were tested using the nonparametric Spearman rank-order correlation with a two-tailed test. For visualization purposes (i.e., to minimize data point overlap from closely situated original targets), jittering was applied by introducing random noise to the observations [30]. Correlations were performed if there were more than 10 studies. We limited the validation analysis to regions adjacent to the primary cortical stimulation site; when effects extended beyond this site (e.g., in the cerebellum), the proximity analysis would not be meaningful given that effects would be driven through indirect (i.e., cortico-cerebellar pathways) stimulation.

## Results

### Symptom-general and symptom-specific meta-analyses

#### TMS is effective across symptom domains

A summary of all standard meta-analysis results can be found in Table 1 and Appendix S5.

**Table 1:** Standard meta-analysis results.

TMS montage	*n* studies; N participants per arm	SMD	95% CI	z	p(z)	I²	τ²
**Across symptoms and protocols**							
Overall effect	91 studies 2269 active; 2014 sham	0.44	[0.30, 0.57]	6.42	<0.00001	77%	0.31
**Negative symptoms**							
*Main effect*	57 studies 1410 active; 1226 sham	0.48	[0.31, 0.65]	5.57	<0.00001	76%	0.30
*subgroup: location*							
L-PFC	37 studies 1045 active; 908 sham	0.62	[0.41, 0.83]	5.77	<0.00001	79%	0.32
L-TPC	6 studies 96 active; 86 sham	0.08	[−0.27, 0.42]	0.42	0.67	20%	0.04
Cerebellum	5 studies 109 active; 102 sham	0.13	[−0.64, 0.90]	0.33	0.74	81%	0.62
*subgroup: frequency*							
>1 Hz	51 studies 1324 active; 1158 sham	0.52	[0.34, 0.70]	5.67	<0.00001	77%	0.32
=1 Hz	6 studies 86 active; 68 sham	0.06	[−0.31, 0.44]	0.32	0.75	21%	0.05
iTBS (L-PFC)	7 studies 151 active; 137 sham	1.01	[0.44, 1.58]	3.49	<0.001	80%	0.46
10 Hz (L-PFC)	21 studies 599 active; 547 sham	0.52	[0.24, 0.80]	3.67	<0.001	80%	0.32
20 Hz (L-PFC)	7 studies 195 active; 171 sham	0.56	[0.11, 1.00]	2.44	0.015	75%	0.27
**Cognitive deficits**							
*Main effect*	15 studies 481 active; 455 sham	0.50	[0.30, 0.71]	4.88	<0.0001	53%	0.08
*subgroup: location*							
L-PFC	12 studies 423 active; 407 sham	0.43	[0.26, 0.60]	4.94	<0.00001	29%	0.03
**Positive symptoms**							
*Main effect*	19 studies 378 active; 333 sham	0.24	[−0.17, 0.64]	1.14	0.25	84%	0.65
*subgroup: location*							
L-TPC	12 studies 222 active; 209 sham	0.44	[0.10, 0.78]	2.51	0.012	64%	0.23
*subgroup: frequency*							
> 1 Hz	6 studies 118 active; 116 sham	0.23	[−0.05, 0.52]	1.59	0.11	0%	0.02
= 1 Hz	13 studies 260 active; 217 sham	0.18	[−0.42, 0.78]	0.60	0.55	89%	1.06
= 1 Hz (L-TPC)	11 studies 190 active; 177 sham	0.49	[0.12, 0.86]	2.59	0.009	63%	0.24

*L-PFC* left prefrontal cortex, *L-TPC* left temporoparietal cortex, *iTBS* intermittent theta burst stimulation.

The standardized mean difference (SMD) across all symptom domains and protocols was 0.44, 95% confidence intervals (CI): [0.30, 0.57], indicating a small but significant improvement for patients receiving active TMS compared to sham (see forest plot of effect sizes in Figure S2). Significant heterogeneity was noted (I² = 77%), and no evidence of publication bias was observed in the funnel plot asymmetry test (p > 0.05, Figure S6A).

Bootstrap analyses yielded SMD estimates nearly identical to those from our random-effects model across all domains and subgroups. Moreover, permutation tests confirmed that the observed significant effects are highly unlikely to have occurred by chance. Detailed results, including bootstrapped SMD values, corresponding 95% CI, and comparisons of the observed pooled SMD against permutation-derived values with two-sided p-values, are provided in Figures S7–S10 and Table S6.

#### TMS is effective in improving negative symptoms

The primary meta-analysis of negative symptoms yielded an SMD of 0.48, 95% CI: [0.31, 0.65], indicating a small yet significant improvement in negative symptoms for patients receiving active TMS compared to sham (see forest plot in Figure S3, Table 1), with moderate-to-high heterogeneity (I² = 76%). The funnel plot in Figure S6B showed no evidence of publication bias.

**Fig. 2:**
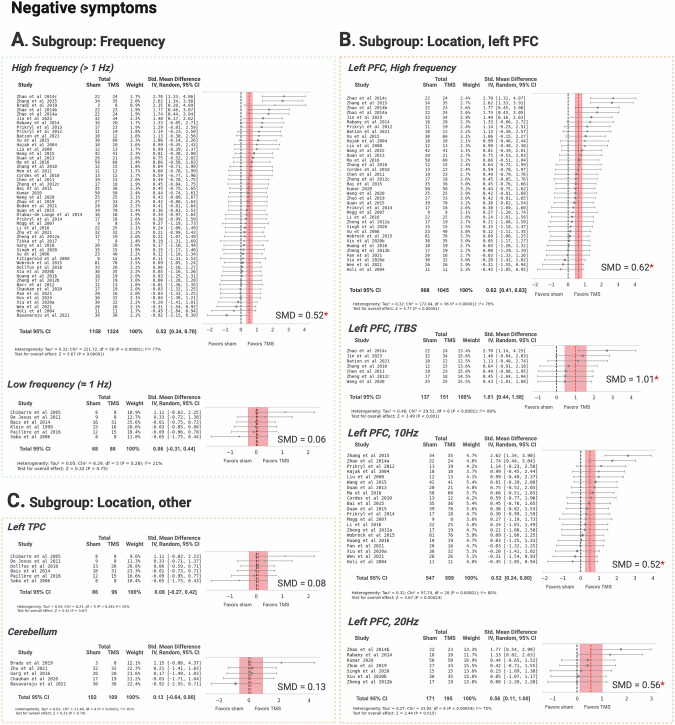
Efficacy of TMS treatment versus sham in negative symptoms of schizophrenia, stratified by stimulation frequency and location. **A** Subgroup by stimulation frequency. **B** Subgroup by stimulation at the left prefrontal cortex. **C** Subgroup by stimulation at other brain regions. PFC prefrontal cortex, TPC temporoparietal cortex, iTBS intermittent theta burst stimulation. Created with BioRender.com.

In the subgroup analyses, we found that high-frequency (HF) TMS demonstrated a significant effect with an SMD of 0.52, 95% CI: [0.34, 0.70], while low-frequency (LF) TMS showed no significant effect (Fig. 2A). Moderate-to-high heterogeneity was observed in the HF subgroup (I² = 77%, Table 1).

Stimulation targeting the left prefrontal cortex (L-PFC) with HF yielded an SMD of 0.62, 95% CI: [0.41, 0.83]. Within this subgroup, iTBS at the L-PFC achieved the highest effect size (SMD = 1.01, 95% CI: [0.44, 1.58]), followed by 20 Hz (SMD = 0.56, 95% CI: [0.11, 1.00]) and 10 Hz (SMD = 0.52, 95% CI: [0.24, 0.80]). High heterogeneity was observed across these subgroups (Fig. 2B). TMS applied to the left temporoparietal cortex (L-TPC) and cerebellum did not yield significant effects, though the small number of studies in these subgroups warrants cautious interpretation (Fig. 2C).

#### TMS is effective in improving cognitive symptoms

The meta-analysis for cognitive symptoms yielded an SMD of 0.50, 95% CI: [0.30, 0.71], indicating a small but significant improvement in cognitive symptoms with active TMS (see forest plot of effect sizes in Figure S4, Table 1). Heterogeneity was moderate (I² = 53%), and the funnel plot in Figure S6C showed no evidence of publication bias (Table 1).

**Fig. 3:**
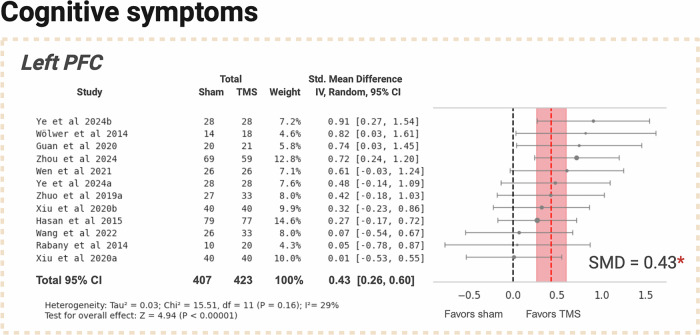
Efficacy of TMS treatment versus sham in cognitive symptoms of schizophrenia, targeting the left prefrontal cortex. PFC prefrontal cortex. Created with BioRender.com.

TMS targeting the L-PFC showed a small effect with an SMD of 0.43, 95% CI: [0.26, 0.60]. Low heterogeneity was noted in this subgroup (I² = 29%, Fig. 3). All studies employed HF protocols, thus a separate subgroup analysis by frequency for this domain was not conducted.

#### Target-specific low frequency TMS is effective in improving positive symptoms

The meta-analysis for positive symptoms did not demonstrate a significant overall effect of TMS (95% CI: [−0.17, 0.64]; see forest plot in Figure S5, Table 1). The funnel plot in Figure S6D indicated no publication bias.

**Fig. 4:**
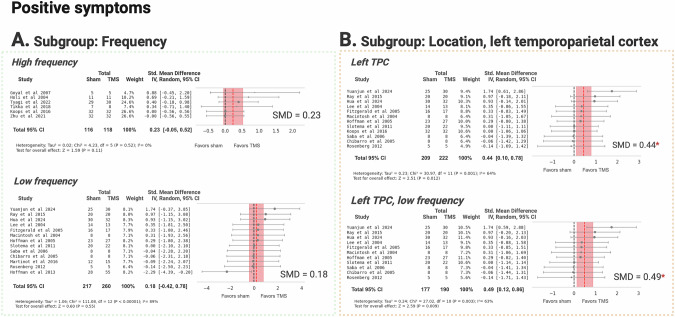
Efficacy of TMS treatment versus sham in positive symptoms of schizophrenia, stratified by stimulation frequency and location. **A** Subgroup by stimulation frequency. **B** Subgroup by stimulation at the left temporoparietal cortex. TPC temporoparietal cortex. Created with BioRender.com.

In addition, HF TMS and LF TMS did not produce a significant effect (Fig. 4A). TMS targeting the L-TPC showed a small but significant effect (SMD = 0.44, 95% CI: [0.10, 0.78]) with moderate heterogeneity (I² = 64%, Table 1). LF TMS at this site resulted in a higher effect (SMD = 0.49, 95% CI: [0.12, 0.86]) with moderate heterogeneity (I² = 63%), indicating consistent effects across studies (Fig. 4B).

### Symptom-general and symptom-specific electric field modeling

#### Site-specific E-field requirements for enhanced symptom-general outcomes

Building on findings from the standard meta-analysis, finite element modeling was applied to correlate E-field distribution with clinical effect size across symptom domains (n = 59 studies), to identify the optimal site defined as the location associated with the highest clinical improvement.

**Fig. 5:**
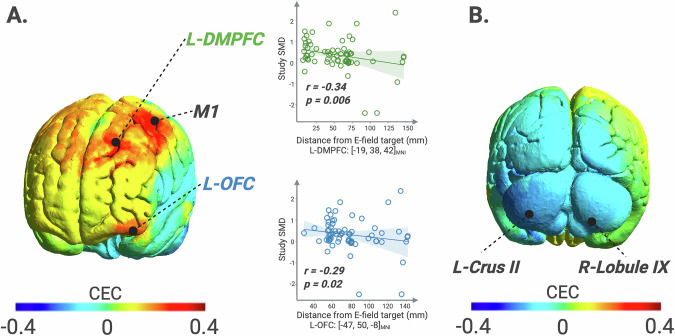
Brain sites associated with the highest TMS-induced clinical improvement across symptom domains. **A** Sites where higher E-field strengths are linked to greater clinical improvement. **B** Sites where lower E-field strengths are linked to greater clinical improvement. L-DMPFC left dorsomedial prefrontal cortex, M1 motor cortex, L-OFC left orbitofrontal cortex, L-Crus left crus, R-Lobule right lobule. Created with BioRender.com.

We found significant positive CEC values in the left motor cortex (MNI: [−44, −15, 64], CEC_MAX_ = 0.40, p = 0.001), left dorsomedial prefrontal cortex (L-DMPFC, MNI: [−19, 38, 42], CEC_MAX_ = 0.40, p = 0.002), and the left orbitofrontal cortex (L-OFC, MNI: [−47, 50, −8], CEC_MAX_ = 0.31, p = 0.02), indicating that higher E-field strengths in these sites are associated with improved clinical outcomes (Fig. 5A). Significant negative CECs were found in the cerebellum at left crus II (MNI: [−36, −73, −44], CEC_MIN_ = −0.30, p = 0.02) and right lobule IX (MNI: [20, −62, −60], CEC_MIN_ = −0.27, p = 0.04), suggesting that lower E-field strengths in these sites could yield better clinical outcomes across symptoms (Fig. 5B).

#### E-field strength in the L-DMPFC and L-OFC linked to improvement in negative symptoms with HF TMS

**Fig. 6:**
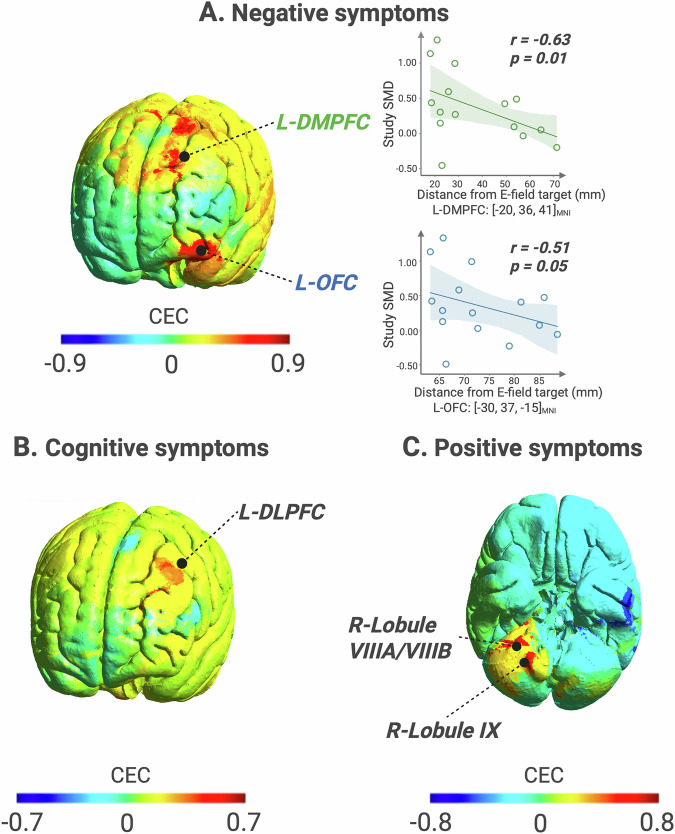
Brain sites associated with the highest TMS-induced clinical improvement for each symptom domain. **A** Negative symptoms. **B** Cognitive symptoms. **C** Positive symptoms. L-DMPFC left dorsomedial prefrontal cortex, L-OFC left orbitofrontal cortex, L-DLPFC left dorsolateral prefrontal cortex, R-Lobule right lobule. Created with BioRender.com.

Given the previous meta-analysis results showing that HF TMS targeting the L-PFC improves negative symptoms in schizophrenia (Section 3.2.), we analyzed the relationship between E-field strength and clinical improvements in studies applying HF TMS to the L-PFC (n = 15 studies) (Fig. 6A). This analysis revealed significant positive CEC values in the L-DMPFC extending to premotor (MNI: [−20, 36, 41], CEC_MAX_ = 0.83, p = 0.0001) and the L-OFC (MNI: [−30, 37, −15], CEC_MAX_ = 0.83, p = 0.0001).

#### E-field strength in the L-DLPFC linked to improvement in cognitive symptoms with HF TMS

Building on our findings that HF TMS targeting the L-PFC significantly reduces cognitive deficits in schizophrenia (Section 3.3.), we further investigated the association between E-field strength and clinical improvement within this subgroup (n = 10 studies) (Fig. 6B). The correlation analysis revealed a significant cluster of positive CEC values in the left dorsolateral prefrontal cortex (L-DLPFC, MNI: [−27, 39, 34], CEC_MAX_ = 0.63, p = 0.04). A pattern of positive CEC values at threshold significance also emerged in the right paravermis in the cerebellum (MNI: [8, −49, −34], CEC_MAX_ = 0.59, p = 0.069).

#### E-field strength in the cerebellum linked to improvement in positive symptoms with LF TMS

Given the previous results showing improvement in positive symptoms with LF TMS targeting the L-TPC (Section 3.4.), we examined the correlation between E-field strength and SMD across these studies (n = 8 studies) for positive symptoms (Fig. 6C). We found statistically significant CEC clusters in the right cerebellar lobules VIIIA (MNI: [32, −38, −50]), VIIIB (MNI: [16, −50, −56]), and IX (MNI: [15, −49, −49]) with CEC_MAX_ = 0.72, p = 0.04 each.

#### Validation of electric-field modeling derived brain targets

We hypothesized that studies whose *actual* targets overlapped with our *predicted* optimal site derived from our E-field modeling approach would be linked to higher clinical improvement.

Across symptoms, proximity to the L-DMPFC and L-OFC sites was significantly associated with larger effect sizes (L-DMPFC: r = −0.34, p = 0.006; L-OFC: r = −0.29, p = 0.02) as illustrated in Fig. 5A.

For negative symptoms, proximity to the optimal L-DMPFC site was strongly linked to better outcomes (r = −0.63, p = 0.01). The L-OFC site demonstrated a similar negative coefficient trending towards significance (r = −0.51, *p* = 0.05), as shown in Fig. 6A.

Due to limited sample size this analysis was not performed for experiments on the cognitive (n = 10 studies) and positive (n = 8 studies) domain.

## Discussion

This study combined meta-analysis and E-field modeling to identify TMS sites for symptom-specific and symptom-general improvements in schizophrenia, pinpointing brain regions associated with symptom relief that support personalized treatment approaches. Across symptom domains, TMS demonstrated a beneficial effect (SMD = 0.44), although responses varied based on target regions and stimulation parameters. In negative symptoms, protocols with HF L-PFC TMS produced a medium effect size (SMD = 0.62), identical to that reported for clozapine (SMD = 0.62) [31]. We note that these comparisons should be interpreted with caution as they do not imply equivalent efficacy or mechanisms and are provided for context for the practicing clinician; direct head‑to‑head trials are needed to compare TMS and clozapine for negative symptoms. Compared to benchmark meta-analyses in the field [32, 33], our study produced an effect size for iTBS at the L-PFC in negative symptoms of SMD = 1.01 [0.44, 1.58], similar to those reported by Tseng et al. (SMD = 1.32 [0.76, 1.88]) and Kishi et al. (SMD = 0.89 [0.55, 1.24]). Slight differences possibly reflect our stricter inclusion criteria, baseline-adjusted analyses using Hedges’ correction for small-sample bias, and the exclusion of studies co-initiating TMS with other therapeutic interventions, in line with our scope of obtaining a homogeneous dataset and robust effect estimates. For cognitive symptoms, protocols with HF L-PFC TMS showed small but significant cognitive gains in schizophrenia (SMD = 0.43), with an effect size higher than that of cognitive remediation (d = 0.29) [34]. For positive symptoms, protocols employing LF TMS to the L-TPC (SMD = 0.49) showed efficacy comparable to the median effect size of antipsychotics (SMD = 0.42) [31], even though direct comparisons remain limited. These findings open the perspective of placing TMS as a complementary approach to traditional antipsychotic and non-pharmacological treatment, with promising effects across symptom domains, particularly for challenging negative and cognitive symptoms. The large effect size observed with iTBS at the L-PFC in negative symptoms further underscores TMS’s potential to advance personalized, domain-targeted interventions in schizophrenia, but more studies are needed.

Building on these symptom-based effects, our analysis also revealed distinct clinical-electric correlations, which could guide E-field dosing adjustments for optimal therapeutic outcomes. Specifically, regarding symptom-general management, stronger E-fields in the left motor cortex, L-DMPFC, and L-OFC correlated with greater symptom improvement, suggesting that higher E-field strengths in these regions may enhance outcomes. Proximity to the L-DMPFC and L-OFC also correlated with better results, offering a first validation of their potential as therapeutic sites. Although left motor cortex dysfunction is linked to both psychomotor retardation [35] and negative symptoms in schizophrenia [36], one pivotal study showed that TMS-induced improvements in negative symptoms, such as anhedonia, do not appear to align with improvements in psychomotor slowing [37]. This suggests that the therapeutic effects of M1-TMS may be mediated by broader modulation of motor, fronto-limbic, and cerebellar networks involved in negative symptom pathophysiology beyond mere normalization of motor dysfunction [38, 39]. Further, we found that a negative clinical-electric correlation in the cerebellum indicated higher effects with lower E-field strengths. This likely reflects the cerebellum’s unique neurofunctional architecture, where an inhibitory cerebellar cortex projects onto excitatory pathways, potentially modulating mesolimbic abnormal activity. In line with this, a reproducible and independently validated neuroimaging marker reflecting an anti-coupling of the cerebellum with the ventral tegmental area was reported to correlate with apathy severity [40] opening the possibility of a circuit-specific approach to TMS, where tailoring stimulation targets and E‑field dosing to distinct symptom-specific circuitry may enhance treatment efficacy [41].

In the negative symptom domain, E-field modeling replicated the L-DMPFC and L-OFC as optimal stimulation sites within the PFC based on maximum E-field strength correlation with clinical improvement (Fig. 6A). Among these sites, the L-DMPFC exhibited the highest positive clinical-electric improvement value. Again, proximity of actual study targets to the L-DMPFC related to improved clinical outcomes, validating this site as a promising therapeutic target for negative symptoms. A similar relationship between actual targets and optimal sites was also found in the L-OFC, albeit at trending significance. Existing literature has highlighted the role of the L-DMPFC and OFC in the pathology of negative symptoms [42–45] and their potential to reduce negative symptom severity through non-invasive stimulation [46]. Mechanistically, the DMPFC plays a role in affect regulation in schizophrenia [47], although more extensively investigated in the context of stimulation treatment for depression [48]. In particular, the DMPFC seems to act as a “dorsal nexus where networks for cognitive control, default-mode rumination, and somatic marker generation converge” [49]. Emerging evidence suggests that DMPFC stimulation may modulate prefrontal-limbic interactions that underlie emotional processing [7] or via an increase in the connectivity of the reward pathways [49–51]. Interestingly, two out of four biotypes of depression responded to DMPFC-TMS, suggesting that specific subgroups, possibly overlapping in part with negative symptom profiles, may benefit from this intervention [52]. On the other hand, the L-OFC is involved in value representation and reward evaluation in schizophrenia [10], functions that are necessary for motivating behavior. Disruptions in these processes can play a role in the pathophysiology of negative symptoms [39, 53] across the bipolar-schizophrenia spectrum [54] making it a plausible target for stimulation. Another perspective suggests that aberrant activation within the lateral orbitofrontal cortex (decoupled from external reward cues) may contribute to the pathophysiology of negative thoughts and emotions in depression [55, 56]. In further support, preliminary studies in schizophrenia have shown promising results in treating negative symptoms with TMS targeting the right OFC with low-frequency protocols [57].

In the cognitive symptom domain, E-field modeling further pinpointed the L-DLPFC as a critical site where higher E-field strengths were significantly associated with cognitive improvements. This finding aligns with existing evidence [58] emphasizing the essential role of the L-DLPFC in cognitive enhancement through TMS in schizophrenia. Additionally, a threshold effect emerged in the cerebellum, which may reflect its involvement in modulating cognitive networks [39, 59], though more studies are needed. Overall, these findings align with recent proposals of individual E-field modeling of TMS-induced effects for personalized dosing [41, 60]. However, given the limited number of studies addressing cognitive symptoms in schizophrenia, further research with larger and more diverse samples is needed to confirm these findings and to refine TMS targeting strategies aimed at improvement in cognitive deficits.

For positive symptoms, E-field modeling of TMS targeting the L-TPC revealed significant positive correlations between stronger E-fields in specific cerebellar regions - namely, right lobules VIIIA, VIIIB and IX - and symptom improvement, particularly with inhibitory protocols. The left TPC is connected to the cerebellum via cortico-cerebellar pathways, which support bidirectional communication, essential for predictive coding and sensory feedback control [61, 62]. Aberrant connectivity in the thalamic-cortico-cerebellar network has been linked to positive symptoms in schizophrenia [63], potentially due to disruptions in sensory feedback processing [64–66]. Lesion network mapping converges with these findings, revealing a common brain circuit defined by functional connectivity to the posterior subiculum of the hippocampus that encompasses the ventral tegmental area and the lobule IX the cerebellum, the latter identified in this work [67]. These disruptions could be partly explained by the altered cerebellum role in forward modeling, i.e., a neural mechanism where the cerebellum continuously generates and updates predictions about sensory input based on incoming information and past experiences, aiming to minimize the error between expectation and reality [68]. In schizophrenia, this predictive mechanism is impaired, leading to misattribution of internally generated thoughts as external stimuli [69, 70] even in early stages of psychosis [71–73].

Our study has several strengths and limitations. A key strength is the use of E-field modeling to directly link TMS-induced electric fields with clinical outcomes, enhancing our understanding of the neuroanatomical correlates of symptom improvement and informing mechanism-based TMS targeting. This approach also enabled E-field dosing recommendations, suggesting lower E-field strengths for cerebellar regions and higher E-field strengths for cortical areas, thereby supporting the development of personalized dosing protocols tailored to neuroanatomy and symptom profiles. Collectively, these findings challenge the “one-site-fits-all” model and underscore the need for targeted, adaptable, E-field dosed TMS strategies to optimize treatment outcomes.

However, there are limitations to consider. A key limitation is that SMD estimates are based on varying numbers of studies, which may affect the precision of effect size comparisons, therefore caution is needed in interpretation. We recommend that future research increase the evidence base for underrepresented stimulation targets (i.e., beyond the traditional approach targeting the prefrontal cortex) to enable more balanced meta-analytic comparisons. Additionally, studies included in our meta-analysis were conducted as adjuncts to antipsychotic therapy in medication-refractory populations, limiting insights into the isolated efficacy of TMS and its application in earlier phases of psychosis. Further research in unmedicated and early-stage populations is warranted to determine whether TMS alone could yield similar benefits. Significant heterogeneity across studies reflects the natural variability in schizophrenia. Further exploration into other sources of heterogeneity, such as differences in study designs, participant characteristics, reported targeting methods and outcome measures, is needed to optimize TMS protocols. In line with this, individual anatomical variations, such as gyrification and cortical thickness, were not captured, although the standardized MNI152 brain template supports group-level reliability [74, 75]. Future efforts can use the same approach in individualized head models. Lastly, while we focused on core TMS parameters, additional protocol variables were not included [76], as they are not currently supported by E-field modeling tools; however, our approach remains clinically relevant for understanding TMS efficacy across and within schizophrenia symptom domains. Future research should investigate these variables, including the impact of accelerated protocols, as the effect sizes observed here may be smaller than those achieved with multiple daily sessions, which have been shown to enhance therapeutic outcomes in depression [77].

In conclusion, this work aimed to identify brain stimulation sites associated with treatment outcomes using an E-field meta-analytical approach by identifying both symptom-general and symptom-specific TMS targets in schizophrenia. These findings represent a step toward establishing personalized neuromodulation and, more broadly, tailored therapeutic interventions in schizophrenia.

## Supplementary information

Supplementary Material

Dataset Positive Symptoms

Dataset Negative Symptoms

Dataset Cognitive Symptoms

## Data Availability

Summary tables of all included studies, organized by symptom domain, are provided in the Supplementary Material. Supplementary Material is available at MP’s website.

## Code availability

Codes for E-field modeling used in this study are openly available, provided by SimNIBS 4.5 (https://simnibs.github.io/simnibs/build/html/index.html). In-house codes to perform the meta-analysis and sensitivity analyses are openly available on GitHub (https://github.com/lorinas/metaModeling).
