# Supplementary material for: Mapping symptom-general and symptom-specific targets for transcranial magnetic stimulation in schizophrenia: an electric-field modeling meta-analysis
Source: Mol Psychiatry. 2025 Sep 22;31(3):1265–75. doi: 10.1038/s41380-025-03238-z (PMC12916294; doi:10.1038/s41380-025-03238-z)
Supplement: Supplementary file 1 — Supplementary Material [file 41380_2025_3238_MOESM1_ESM.docx]

**SUPPLEMENTARY INFORMATION**

**Appendix S1.** Search Strategy and Data Extraction Process

**Appendix S2.** List of Excluded Studies From the Standard Meta-Analysis

**Appendix S3.** Supplementary Methods: Bootstrapping Sensitivity Analysis and Permutation Testing

**Appendix S4.** List of Excluded Studies From the FEM Simulations

**Appendix S5.** Nonsignificant Findings

**Figure S1.** PRISMA algorithm

**Figure S2.** Forest Plot of Effect Sizes (SMD) of TMS in Schizophrenia across all symptoms

**Figure S3.** Forest Plot of Effect Sizes (SMD) of TMS Targeting Negative Symptoms in Schizophrenia

**Figure S4.** Forest Plot of Effect Sizes (SMD) of TMS Targeting Cognitive Symptoms in Schizophrenia

**Figure S5.** Forest Plot of Effect Sizes (SMD) of TMS Targeting Positive Symptoms in Schizophrenia

**Figure S6.** Funnel Plot Analysis for Publication Bias Assessment Across Symptom-General and Symptom-Specific Domains of Schizophrenia

**Figure S7.** Distributions of bootstrapped and permutation pooled standardized mean differences (SMD) for four symptom groups (All, Negative, Cognitive, Positive)

**Figure S8.** Distributions of bootstrapped and permutation pooled standardized mean differences (SMD) for negative symptom subgroups

**Figure S9.** Distributions of bootstrapped and permutation pooled standardized mean differences (SMD) for cognitive symptom subgroup

**Figure S10.** Distributions of bootstrapped and permutation pooled standardized mean differences (SMD) for positive symptom subgroups

**Table S1.** Study Overview of Included Randomized Controlled Trials in Negative Symptoms

**Table S2.** Study Overview of Included Randomized Controlled Trials in Cognitive Symptoms

**Table S3.** Study Overview of Included Randomized Controlled Trials in Positive Symptoms

**Table S4.** Risk-of-Bias Assessments

**Table S5.** Breakdown of Sample Sizes Across All Included Studies

**Table S6**. Pooled Standardized Mean Differences (SMD) Across Symptom Domains Using Random Effects, Bootstrapping, and Permutation Testing

**References**

**Appendix S1.** Search strategy and data extraction process

1. **Outcome Measurement Instruments to Assess Negative, Cognitive, and Positive Symptoms**

For negative symptoms, scales such as the Positive and Negative Syndrome Scale Negative Subscale (PANSS-N) and the Scale for the Assessment of Negative Symptoms (SANS) were employed.

Cognitive symptoms were assessed using the Brief Assessment of Cognition in Schizophrenia (BACS) composite score, Repeatable Battery for the Assessment of Neuropsychological Status (RBANS) total score, Neurocognitive Composite Score, Montreal Cognitive Assessment (MoCA), Cognitive Stability Index (CSA), Facial Affect Recognition (PFA) and N-back task accuracy (active) assessment score.

Positive symptoms were evaluated using the Schizophrenia Hallucination Rating Scale (SHRS), Hallucination Change Scale (HCS), Positive and Negative Syndrome Scale Positive Subscale (PANSS-P), Auditory Hallucinations Rating Scale (AHRS), and the Scale for the Assessment of Positive Symptoms (SAPS).

1. **Search Strings Related to Schizophrenia and TMS Used for the Other Databases**

**PubMed:** 926 hits

(“schizophreni*” OR "schizoaffective disorder" OR "schizophreniform disorder" OR "schizophrenia"[MeSH Terms] OR “CHR” OR “Clinical High Risk” OR “Ultra High Risk” OR “UHR” OR "Psychotic Disorders"[MeSH Terms] OR "Psychotic Disorder*") AND ("transcranial magnetic stimulation" OR "TMS" OR "rTMS" OR "theta burst" OR "iTBS" OR “cTBS” OR "transcranial Magnetic Stimulation*"[MeSH Terms])

**EMBASE:** 1,039 hits

('schizophreni*':ab,kw,ti OR 'schizoaffective disorder':ab,ti,kw OR 'schizophreniform disorder':ab,kw,ti OR 'schizophrenia spectrum disorder'/exp OR 'psychotic disorder*':ab,kw,ti OR ‘CHR’:ab,kw,ti OR ‘Clinical High Risk’:ab,kw,ti OR ‘Ultra High Risk’:ab,kw,ti OR ‘UHR’:ab,kw,ti) AND ('transcranial magnetic stimulation':ab,kw,ti OR 'tms':ab,kw,ti OR 'rtms':ab,kw,ti OR 'theta burst':ab,kw,ti OR 'itbs':ab,kw,ti OR 'cTBS':ab,kw,ti OR 'transcranial magnetic stimulation'/exp) AND ('article'/it OR 'article in press'/it OR 'review'/it)

**PsycINFO:** 864 hits

(schizophreni* OR "schizoaffective disorder" OR "schizophreniform disorder" OR "Psychotic Disorder*" OR “CHR” OR “Clinical High Risk” OR “Ultra High Risk” OR “UHR”) AND ("transcranial magnetic stimulation" OR "TMS" OR "rTMS" OR "theta burst" OR "iTBS" OR “cTBS”)

Source type: Scholarly Journals

**Web of Sciences (Web of Science, Core Database Collection):** 1,482 hits

(schizophreni* OR "schizoaffective disorder" OR "schizophreniform disorder" OR "Psychotic Disorder*" OR “CHR” OR “Clinical High Risk” OR “Ultra High Risk” OR “UHR”) AND ("transcranial magnetic stimulation" OR "TMS" OR "rTMS" OR "theta burst" OR "iTBS" OR “cTBS”)

Search type: “Topic”. Document type: Article, review, proceedings paper, early access

15 studies were sourced from the meta-analysis by Wang et al. [1] and 8 studies from the meta-analysis by Lorentzen et al [2].

In total: 4,311 hits. After removal of 2,023 duplicates: 2,288 final hits

1. **Data extraction**

KP and AFD extracted the following information from each included study: author names, publication year, country, study type, number of participants, average participant age, sex distribution, diagnosis, primary and secondary outcomes, side effects, frequency and intensity of TMS (including the total number of stimuli and treatments), motor threshold percentage (MT%), TMS target, use of neuro-navigation, method of targeting (scalp-based, anatomical, EEG landmarks, Montreal Neurological Institute (MNI) coordinates in mm [x, y, z]), nature of the sham intervention, outcome measure (rating scale), and the post-treatment at first follow-up scores.

1. **Risk of bias assessment**

We assessed studies for bias across five domains (articles in non-English languages were not evaluated) using the Cochrane Risk of Bias Tool 2.0 (<https://methods.cochrane.org/risk-bias-2>): (A) Randomization process (including allocation sequence generation and concealment), (B) Deviations from intended interventions (bias due to non-protocol interventions), (C) Missing outcome data (dropouts), (D) Outcome measurement (using a validated tool), and (E) Selection of the reported result (alignment with the protocol and methods section). According to the Cochrane Risk of Bias Tool 2.0 guidelines, the highest risk score assigned in any domain determined the overall risk of bias score for each study (Table S4).

**Appendix S2. List of Excluded Studies from the Standard Meta-Analysis**

**1.** **Studies not employing TMS**

**Negative Symptoms (N=3 studies):**

Kos et al. (2024b) doi: 10.1016/j.psychres.2024.115743

Lisoni et al. (2022) doi: [10.1016/j.jpsychires.2022.09.011](https://doi.org/10.1016/j.jpsychires.2022.09.011)

Palm et al. (2016) doi: 10.1093/schbul/sbw041

**Cognitive Symptoms (N=1 study):**

Jeon et al. (2018) doi: 10.1016/j.schres.2017.12.009

**Positive Symptoms (N=4 studies):**

Brunelin et al. (2012) doi: 10.1176/appi.ajp.2012.11071091

Chang et al. (2018) doi: 10.1093/ijnp/pyy071

Fitzgerald et al. (2014) doi: 10.1016/j.brs.2014.07.036

Koops et al. (2018) doi: 10.1016/j.schres.2018.06.058

**2.** **Studies with no full report of baseline/endpoint scores**

**Negative Symptoms (N=13 studies):**

Gan et al. (2014a) doi: PMID: 27337795

Gan et al. (2014b) doi: PMID: 27337795

Gan et al. (2015) doi: PMID: 27337795

Gan et al. (2021) doi: PMID: 10.1016/j.psychres.2021.113876

Goyal et al. (2007) doi: [10.1176/jnp.2007.19.4.464](https://doi.org/10.1176/jnp.2007.19.4.464)

Güleken et al. (2020) doi: [10.1016/j.scog.2020.100183](https://doi.org/10.1016/j.scog.2020.100183)

Novak et al. (2006) doi: PMID: 16648775

Prikryl et al. (2007) doi: [10.1016/j.schres.2007.06.019](https://doi.org/10.1016/j.schres.2007.06.019)

Rosa et al. (2007) doi: [10.4088/jcp.v68n1009](https://doi.org/10.4088/jcp.v68n1009)

Schneider et al. (2008a) doi: [10.1016/j.jpsychires.2005.02.008](https://doi.org/10.1016/j.jpsychires.2005.02.008)

Schnieder et al. (2008b) doi: [10.1016/j.jpsychires.2005.02.008](https://doi.org/10.1016/j.jpsychires.2005.02.008)

Wen-Xiang et al. (2012) doi: [10.1016/j.neulet.2014.10.029](https://doi.org/10.1016/j.neulet.2014.10.029)

Quan et al. (2015a) doi: 10.1016/j.neulet.2014.10.029

**Cognitive Symptoms (N=1 study):**

Voineskos et al. (2021) doi: 10.1016/j.bpsc.2020.11.011

**Positive Symptoms (N=1 study):**

Vercammen et al. (2009) doi: 10.1176/appi.neuropsych.21.3.260

**3.** **Studies with primary outcome in another domain**

**Negative Symptoms (N=2 studies):**

Guan et al. (2020) doi: 10.1038/s41398-020-0745-6, primary outcome is cognitive dysfunction.

Rosenberg et al. (2012) doi: [10.1186/1744-859X-11-13](https://doi.org/10.1186/1744-859x-11-13), primary outcome is auditory hallucinations.

**Appendix S3. Supplementary Methods: Bootstrapping Sensitivity Analysis and Permutation Testing**

We applied a bootstrapping sensitivity analysis to assess the robustness of the pooled SMD in the presence of outliers, and a standard nonparametric permutation test to evaluate its significance under the null hypothesis of no effect. Bootstrapping is a statistically principled sensitivity method that empirically estimates the sampling distribution of the pooled effect size without discarding any data. By repeatedly resampling the original set of studies with replacement (1’000 iterations), we quantified the influence of each study, including those with high variance, on the overall effect estimate and derived robust 95% confidence intervals that reflect true sampling variability. In contrast, excluding studies solely on the basis of a predefined threshold (e.g.,  > 2.5) could reduce sample size and statistical power, and risks introducing selection bias, given that such exclusion may ignore the possibility that high‑variance studies may *legitimately* reflect clinical heterogeneity rather than mere measurement error. Specifically, in each of 1’000 bootstrap iterations, the pooled SMD was re-calculated using updated weights (based on re-calculated variances). The distribution of these bootstrap estimates was then used to derive the mean, standard error, and a percentile-based 95% confidence interval for the pooled SMD.

We also conducted a standard nonparametric permutation test procedure to evaluate the significance of the observed pooled SMD under the null hypothesis of no effect. For each of the permutation iterations, the sign of each study’s SMD was randomly flipped, and the pooled SMD was recomputed using the same random-effects model. The resulting permutation distribution was used to calculate a two-sided p-value by comparing the absolute value of the observed pooled SMD against the permutation-derived values.

**Appendix S4. List of Excluded Studies from the FEM Simulations**

**1.** **Studies not retained due to no report of coil type**

**Negative Symptoms (N=13 studies):**

Bai et al. (2015) doi: Chin. J. Health Psychol. 2015;23:649–653

Duan et al. (2013) doi: [10.1038/s41537-022-00248-6](https://doi.org/10.1038%2Fs41537-022-00248-6)

Liu et al. (2008) doi: [10.3969/j.issn.1672-0458.2008.03.002](http://dx.chinadoi.cn/10.3969/j.issn.1672-0458.2008.03.002)

Ma et al. (2016) doi: [10.3877/cma.j.issn.1674-0785.2016.14.007](http://dx.chinadoi.cn/10.3877/cma.j.issn.1674-0785.2016.14.007)

Ren et al. (2011) doi: [10.3969/j.issn.1000-6729.2011.02.003](http://dx.chinadoi.cn/10.3969/j.issn.1000-6729.2011.02.003)

Tikka et al. (2017) doi: 10.1097/YCT.0000000000000343

Wang et al. (2015) doi: [10.11919/j.issn.1002-0829.217024](https://doi.org/10.11919%2Fj.issn.1002-0829.217024)

Wang et al. (2020) doi: [10.1016/j.schres.2019.12.008](https://doi.org/10.1016/j.schres.2019.12.008)

Xu et al. (2006) doi: [10.3321/j.issn:1673-8225.2006.46.012](http://dx.chinadoi.cn/10.3321/j.issn:1673-8225.2006.46.012)

Xu et al. (2015) doi: NA

Zhang et al. (2010) doi: [10.3969/j.issn.1002-0829.2010.05.002](http://dx.chinadoi.cn/10.3969/j.issn.1002-0829.2010.05.002)

Zhang et al. (2015) doi: [10.3969/j.issn.1672-9463.2015.09.008](http://dx.chinadoi.cn/10.3969/j.issn.1672-9463.2015.09.008)

Zhou et al. (2024) doi: 10.1016/j.psychres.2023.115672

**2.** **Studies not retained due to no clear report of targeting, inferred coordinates or intensity**

**Negative Symptoms (N=10 studies):**

Bodén et al. (2021) doi: 10.1016/j.jad.2021.04.053

Li et al. (2016) doi: [10.1016/j.psychres.2016.04.046](https://doi.org/10.1016/j.psychres.2016.04.046)

Prikryl et al. (2012) doi: [10.1017/S1461145712000508](https://doi.org/10.1017/S1461145712000508)

Jin et al. (2023) doi: 10.1016/j.psychres.2022.115033

Zhao et al. (2014a) doi: 10.3969/j.issn.1002-0829.2014.01.003

Zhao et al. (2014b) doi: 10.3969/j.issn.1002-0829.2014.01.003

Zhao et al. (2014c) doi: 10.3969/j.issn.1002-0829.2014.01.003

Zheng et al. (2012a) doi: PMID: 23073583

Zheng et al. (2012b) doi: PMID: 23073583

Zheng et al. (2012c) doi: PMID: 23073583

**Positive Symptoms (N=1 study):**

Hua et al. (2024) doi: 10.1001/jamanetworkopen.2024.44215

**3.** **Studies not retained due to coil type not supported in SimNIBS**

**Negative Symptoms (N=5 studies):**

De Jesus et al. (2011) doi: [10.1016/j.psychres.2010.11.022](https://doi.org/10.1016/j.psychres.2010.11.022) (Neurosoft Ltd.)

Huang et al. (2016) doi: [10.11919/j.issn.1002-0829.216044](https://doi.org/10.11919/j.issn.1002-0829.216044) (YRDCCY-I)

Klein et al. (1999) doi: [10.1016/s0006-3223(99)00182-1](https://doi.org/10.1016/s0006-3223(99)00182-1) (Cadwell)

Quan et al. (2015b) doi: [10.1016/j.neulet.2014.10.029](https://doi.org/10.1016/j.neulet.2014.10.029) (Cadwell)

Wen et al. (2021) doi: PMID: 27337795 (YRDCCY-I)

**Cognitive Symptoms (N=1 study):**

Wen et al. (2021) doi: PMID: 27337795 (YRDCCY-I)

**Positive Symptoms (N=2 studies):**

Lee et al. (2004) doi: [10.1016/j.neulet.2004.11.048](https://doi.org/10.1016/j.neulet.2004.11.048) (Neotonus)

Yuanjun et al. (2024) doi: 10.1038/s41398-024-03106-4 (YIRUIDE YCD-I, Wuhan, China)

**Appendix S5.** Nonsignificant Findings

1. **Meta-analysis of TMS effects on negative symptoms of schizophrenia**
   1. **Low-frequency TMS (1 Hz) stimulation sub-group**

Six sham-controlled studies delivered low-frequency stimulation TMS (1 Hz) targeting negative symptoms in schizophrenia via stimulation of the left prefrontal cortex (L-PFC) and left temporo-parietal cortex (L-TPC). The confidence interval was wide: 95% CI: -0.31 to 0.44 with no significant effect size over TMS (p=0.75).

- 1. **Left temporo-parietal target sub-group**

Six sham-controlled studies investigated the effects of TMS on the negative symptoms of schizophrenia by targeting the left temporo-parietal cortex (L-TPC).The confidence interval was wide: 95% CI: -0.27 to 0.42 with no significant effect size over TMS (p=0.67).

- 1. **Cerebellum target sub-group**

Five sham-controlled studies investigated the effects of TMS on the negative symptoms of schizophrenia by targeting vermis in the cerebellum. The confidence interval was wide: 95% CI : -0.64 to 0.90 with no significant effect size over TMS (p=0.74).

1. **Meta-analysis of TMS effects on positive symptoms of schizophrenia**
   1. **Main group**

Nineteen sham-controlled studies targeted positive symptoms in schizophrenia irrespective of target and protocol. The confidence interval was wide: 95% CI: -0.17 to 0.64 with no significant effect size over TMS (p=0.25).

- 1. **Low-frequency TMS (1 Hz) stimulation sub-group**

Thirteen sham-controlled studies delivered low-frequency stimulation TMS (1 Hz) targeting positive symptoms in schizophrenia. The confidence interval was wide: 95% CI: -0.42 to 0.78 with no significant effect size over TMS (p=0.55).

- 1. **High-frequency TMS (>1 Hz) stimulation sub-group**

Six sham-controlled studies delivered high-frequency stimulation TMS targeting positive symptoms in schizophrenia. The confidence interval was wide: 95% CI: -0.05 to 0.52 with no significant effect size over TMS (p=0.11).

**Association between optimal targets and actual study sites**

- 1. **Left motor (M1) optimal target across symptom domains**

The association between treatment response and the distance of actual TMS study sites to the optimal target in the left motor across symptom domains was not significant (r = 0, p = 0.98 ).

- 1. **Left premotor optimal target in negative symptoms**

The association between treatment response and the distance of actual TMS study sites to the optimal target in the left premotor in the negative symptom domain was not significant (r = -0.27, p = 0.3).


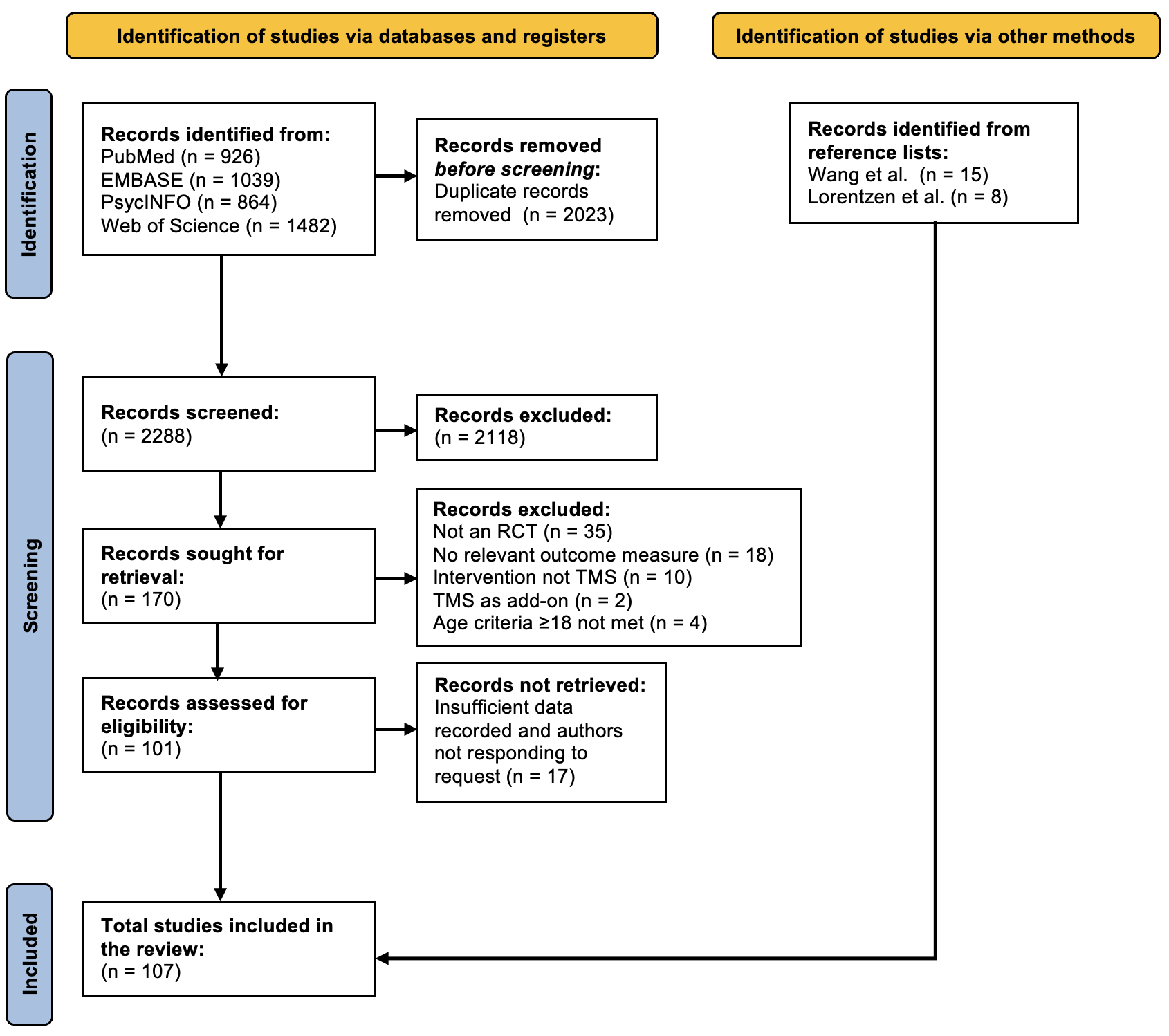


Figure S1. Preferred Reporting Items for Systematic Reviews and Meta-Analyses (PRISMA) flow chart. Abbreviations: EMBASE = Excerpta Medica Database; PsycINFO = Psychological Information Database; Web of Science = Web of Science, Core Database Collection.

**
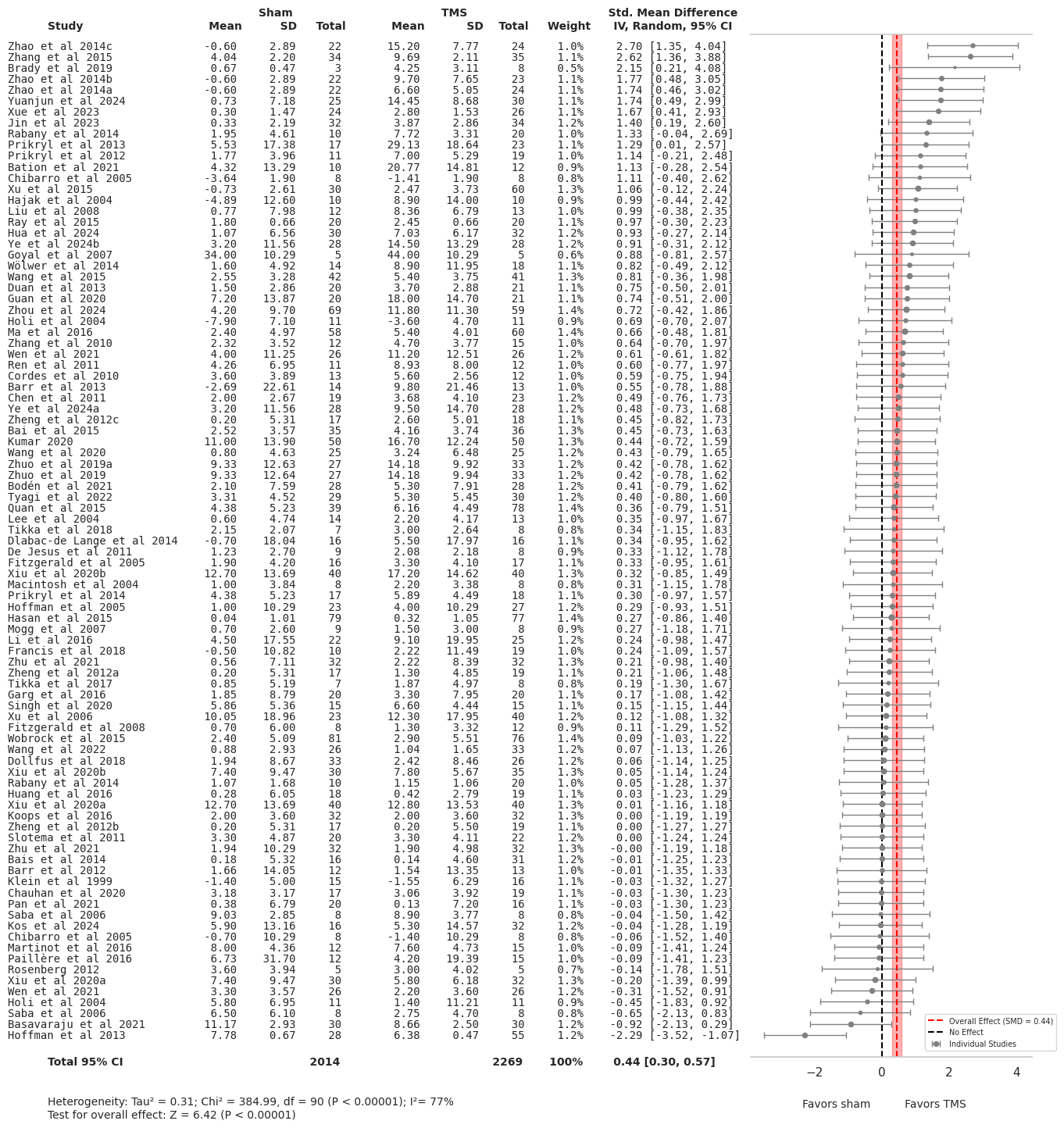
**

Figure S2. Forest Plot of Effect Sizes (SMD) of TMS in Schizophrenia Across all Symptoms

**
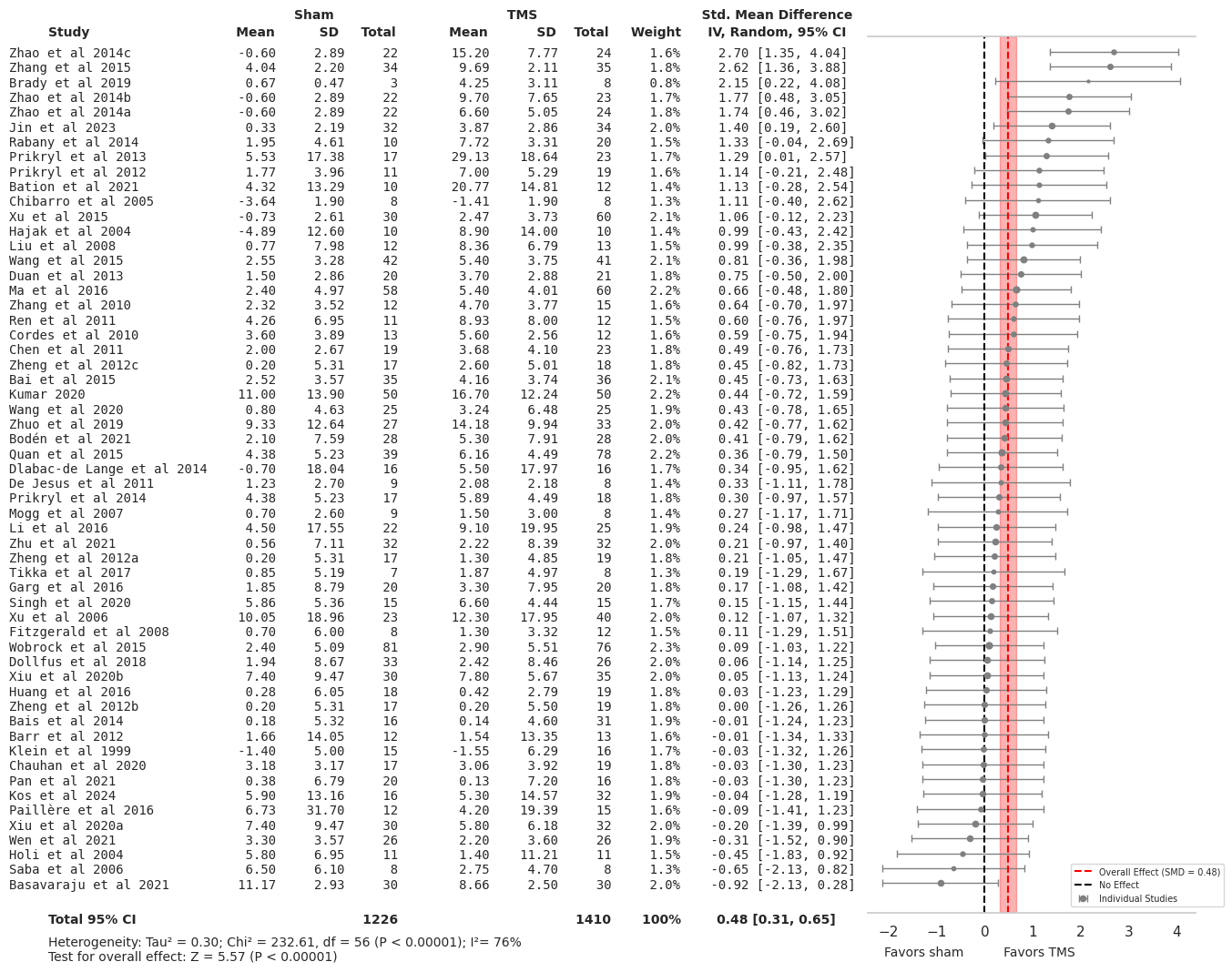
**

Figure S3. Forest Plot of Effect Sizes (SMD) of TMS Targeting Negative Symptoms in Schizophrenia

**
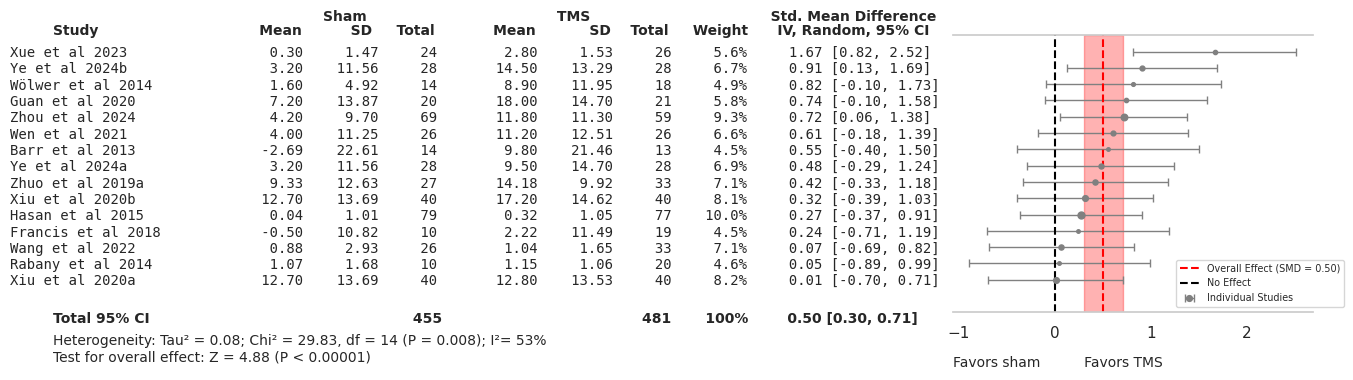
**

Figure S4. Forest Plot of Effect Sizes (SMD) of TMS Targeting Cognitive Symptoms in Schizophrenia


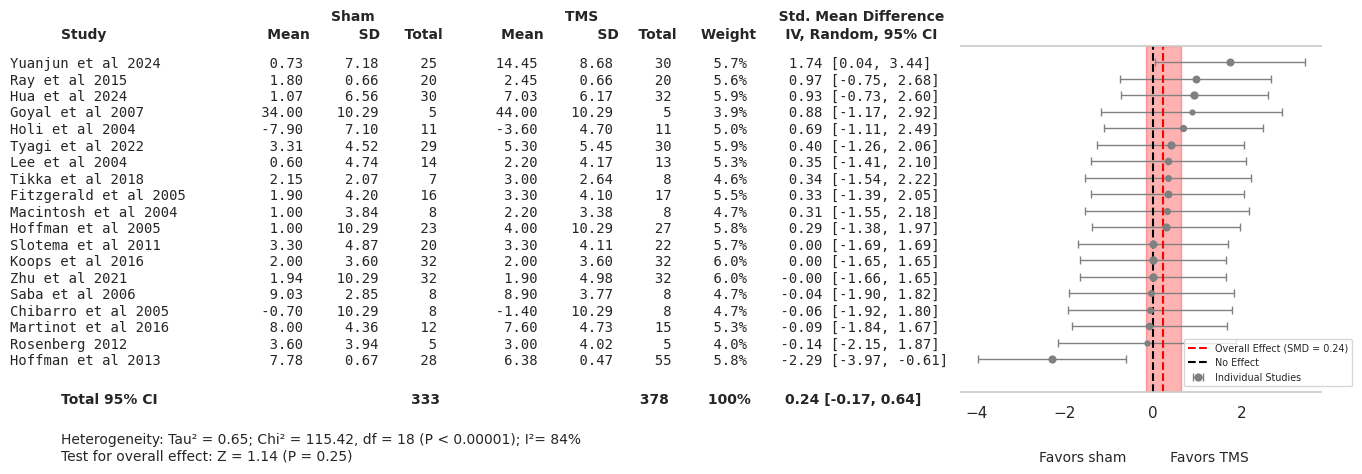


Figure S5. Forest Plot of Effect Sizes (SMD) of TMS Targeting Positive Symptoms in Schizophrenia

**
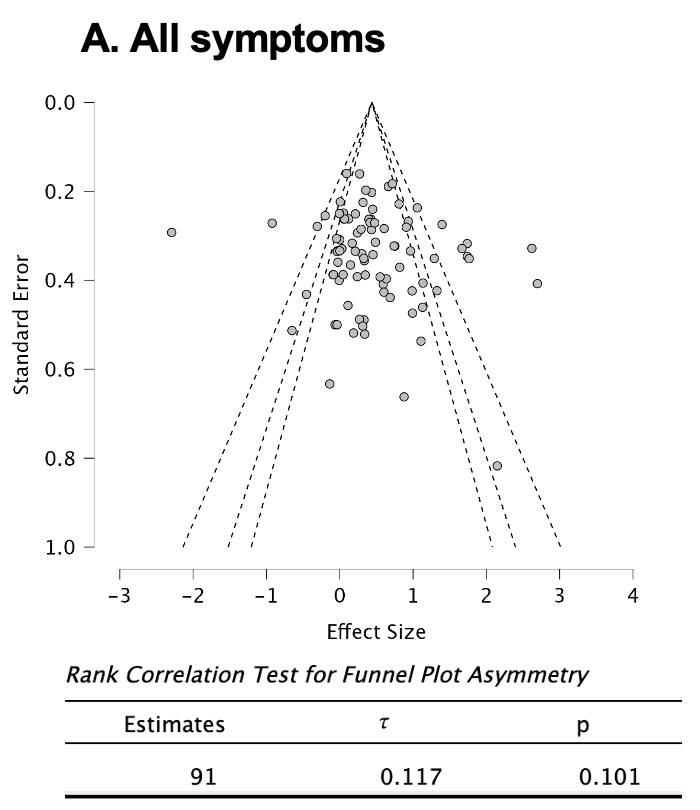
**

**
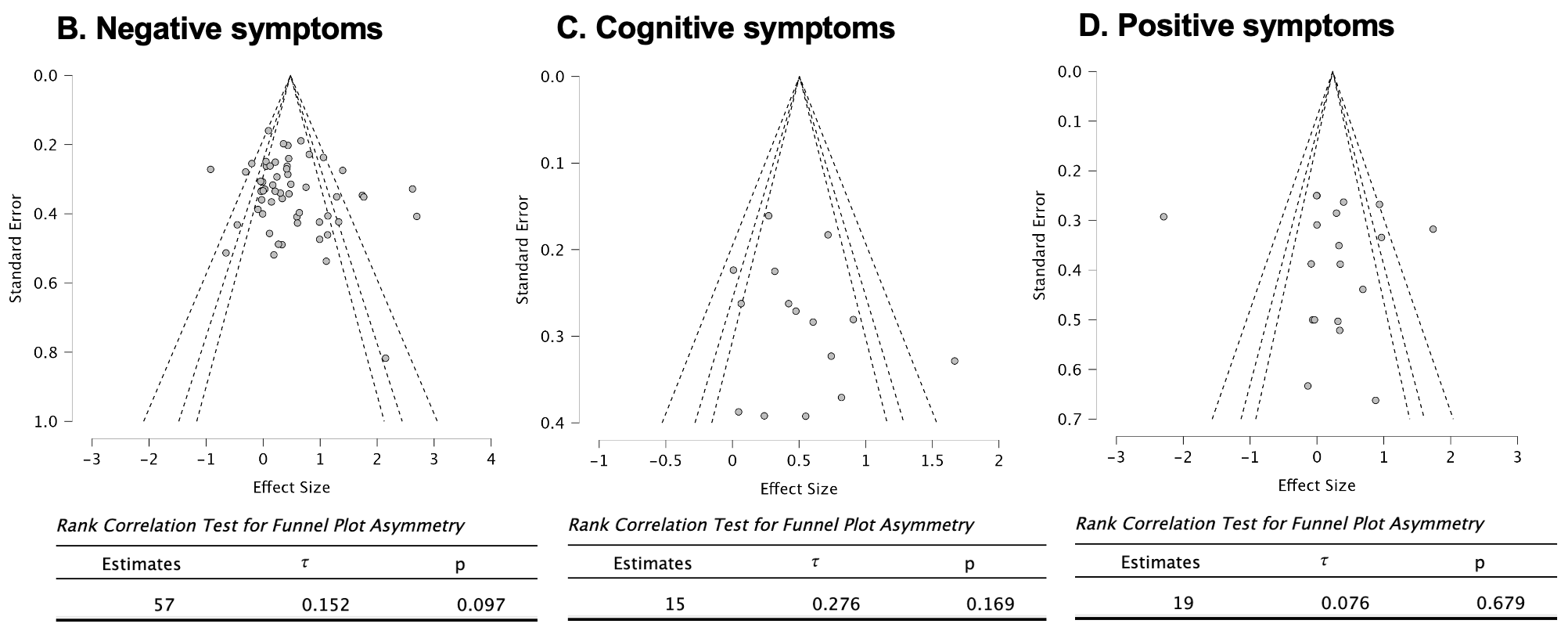
**

Figure S6. Funnel Plot Analysis for Publication Bias Assessment in Symptom-General and Symptom-Specific Domains of Schizophrenia. Each plot compares the standard error against the effect size of studies. The dashed lines represent the expected distribution of studies in the absence of bias. Points falling outside these lines suggest potential asymmetry, indicating possible bias or heterogeneity. The asymmetry in the plot is evaluated using Kendall’s rank correlation test.


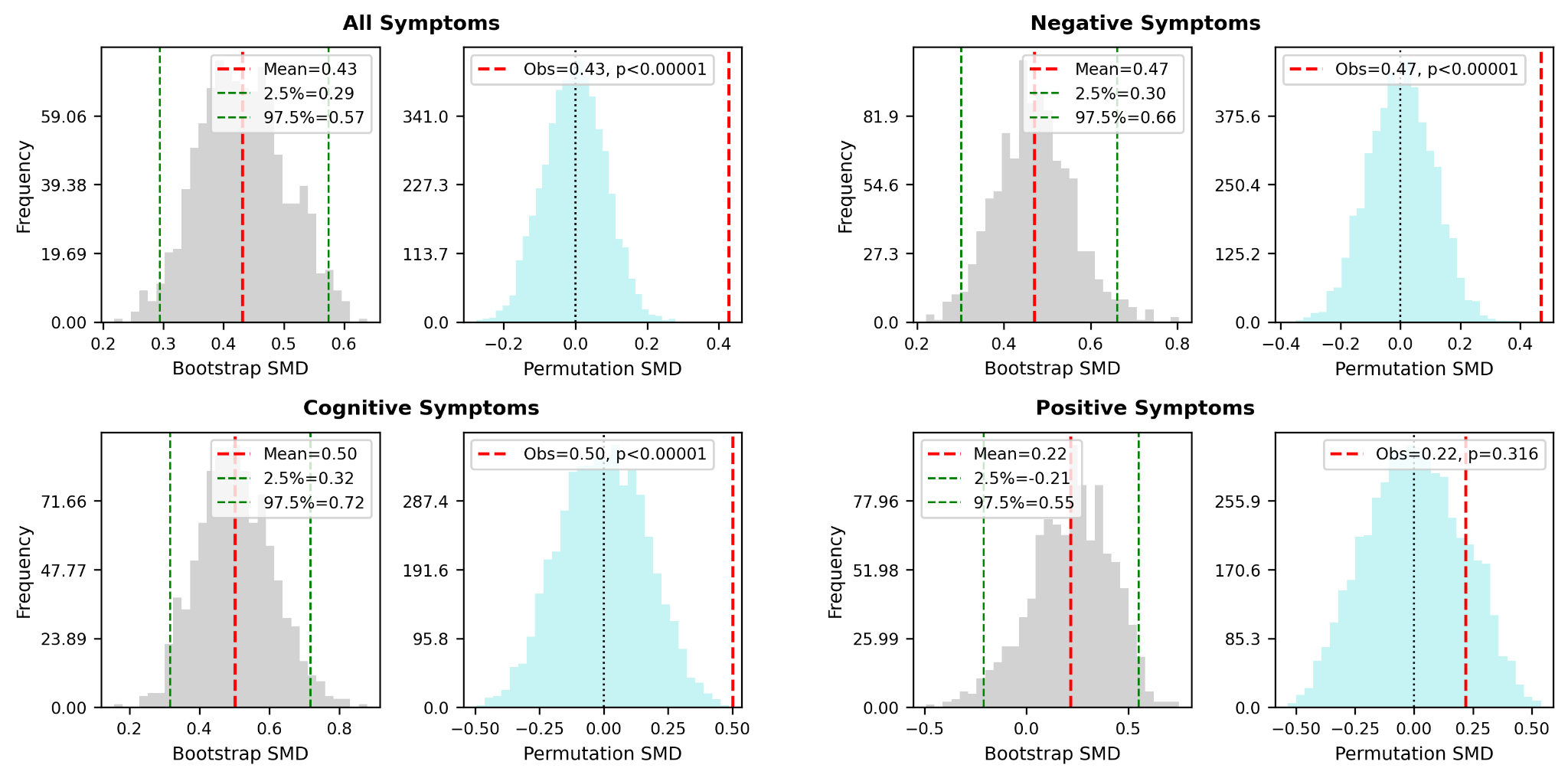


Figure S7. Distributions of bootstrapped and permutation pooled standardized mean differences (SMD) for the symptom groups (All, Negative, Cognitive, Positive): In the left panels, the histogram of bootstrapped estimates (n = 1,000) displays the red dashed line indicating the mean pooled SMD, while the green dashed lines mark the 2.5th and 97.5th percentiles (95% confidence interval). In the right panels, the histogram of pooled SMD estimates obtained from 5,000 permutation (sign-flip) iterations is presented. The red dashed line denotes the observed pooled SMD and its corresponding p-value, whereas the black dotted vertical line at SMD = 0 serves as a reference for the null hypothesis of no effect.


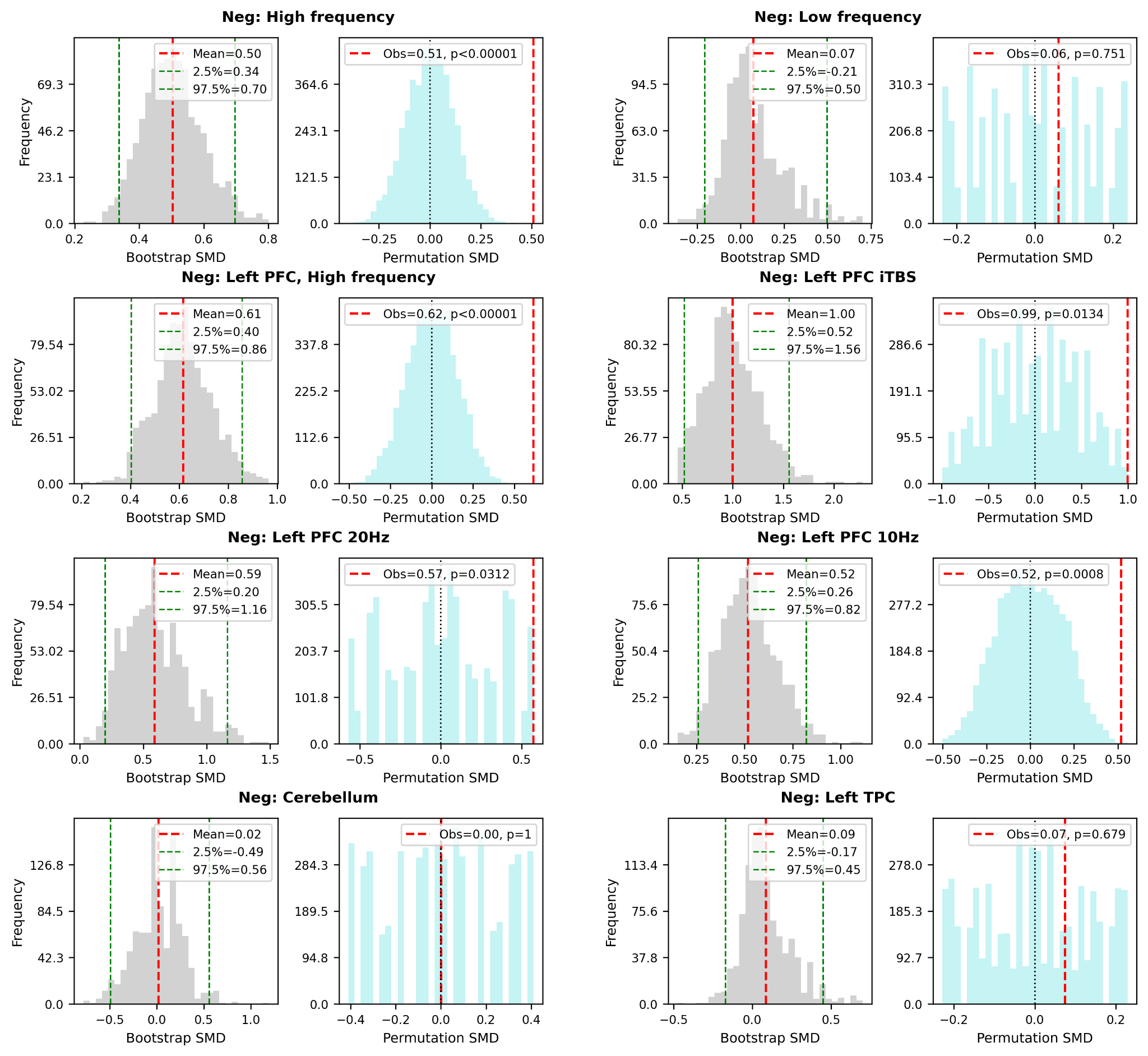


Figure S8. Distributions of bootstrapped and permutation pooled standardized mean differences (SMD) for negative symptom subgroups


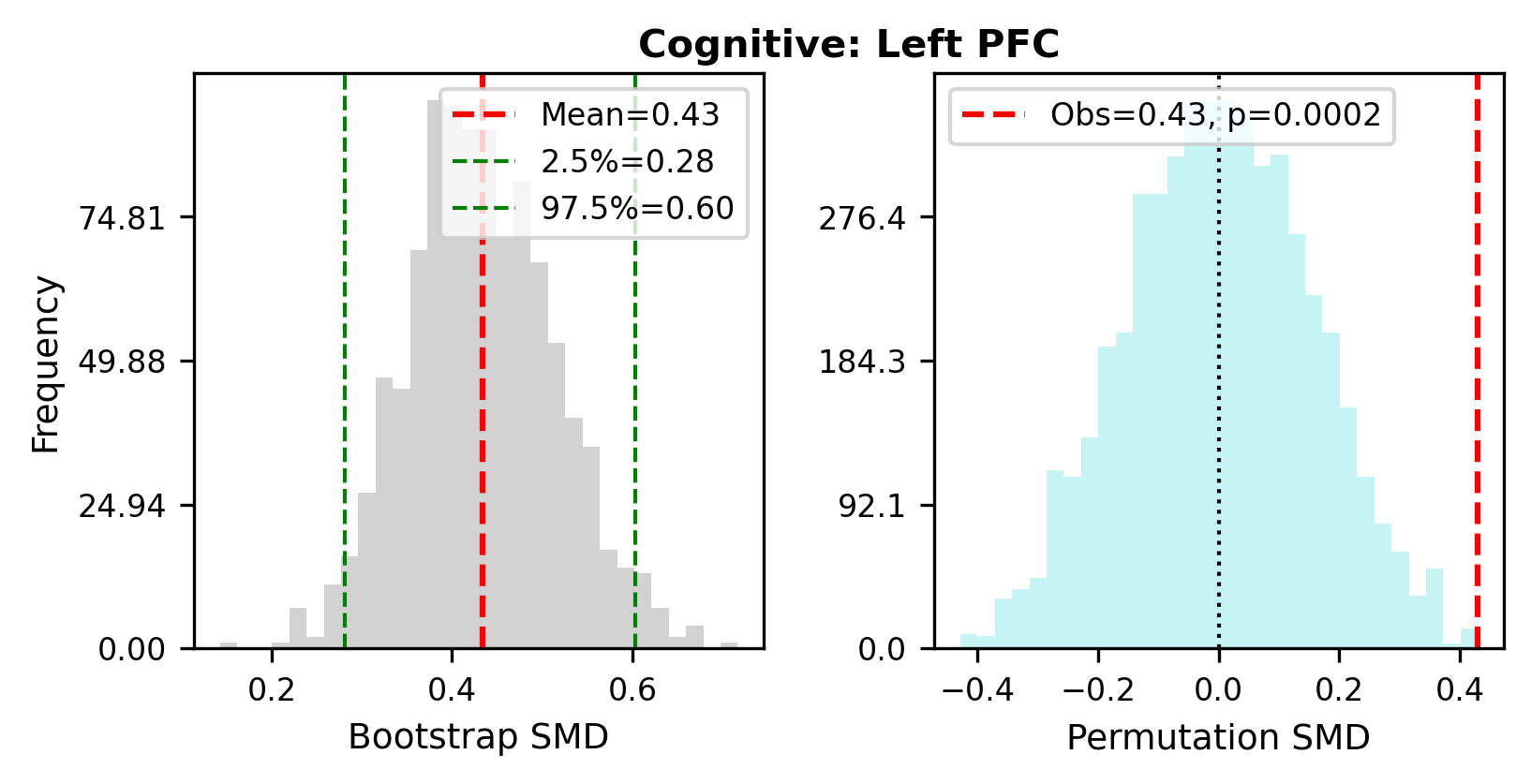


Figure S9. Distributions of bootstrapped and permutation pooled standardized mean differences (SMD) for cognitive symptom subgroup


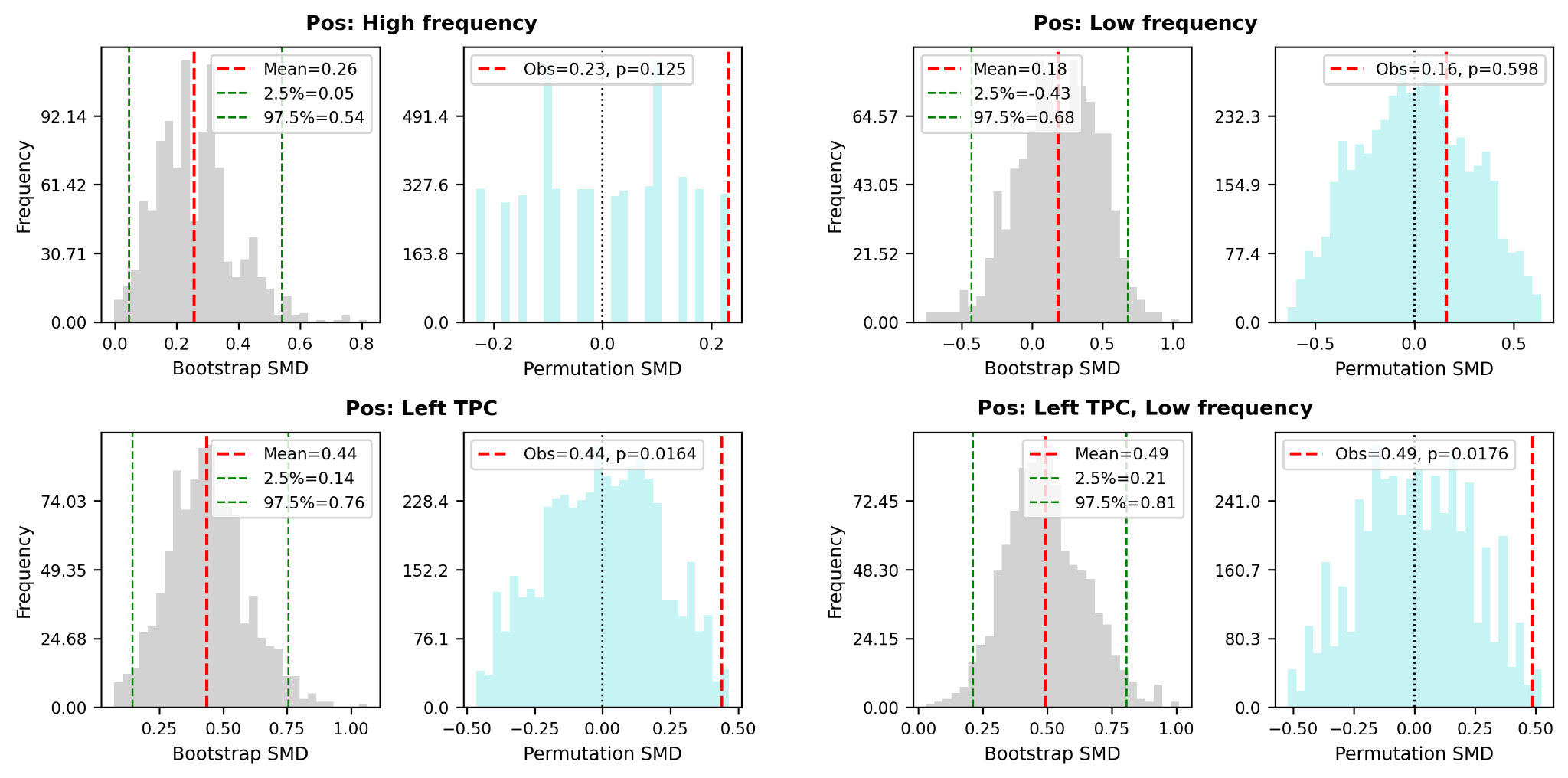


Figure S10. Distributions of bootstrapped and permutation pooled standardized mean differences (SMD) for positive symptom subgroups

Table S1. Study Overview of Included Randomized Controlled Trials in Negative Symptoms


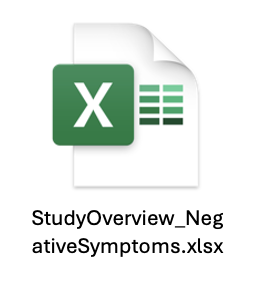


Table S2. Study Overview of Included Randomized Controlled Trials in Cognitive Symptoms


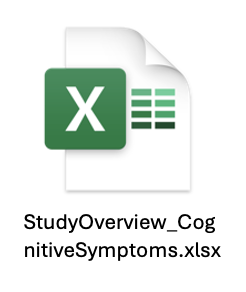


Table S3. Study Overview of Included Randomized Controlled Trials in Positive Symptoms


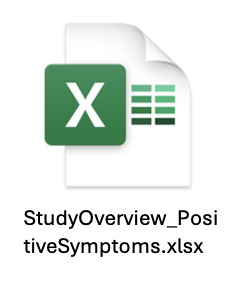


**Table S4. Risk-of-Bias Assessments**

| **NEGATIVE SYMPTOMS: Study** | **Random sequence generation (selection bias)** | **Allocation concealment (selection bias)** | **Blinding of participants and personnel (performance bias)** | **Blinding of outcome assessment (detection bias)** | **Incomplete outcome data (attrition bias)** | **Selective reporting (reporting bias)** | **Other bias** |
| --- | --- | --- | --- | --- | --- | --- | --- |
| **Bai et al 2015** | Low | Some concerns | Low | Some concerns | Some concerns | Low | Low |
| **Bais et al 2014** | Low | Low | Low | Low | Low | Low | Low |
| **Barr et al 2012** | Low | Low | Low | Low | Low | Low | Low |
| **Basavaraju et al 2021** | Low | Low | Low | Low | Some concerns | Low | Low |
| **Bation et al 2021** | Low | Low | Some concerns | Low | Low | Low | Some concerns |
| **Bodén et al 2021** | Low | Some concerns | High | Some concerns | Low | Some concerns | Low |
| **Brady et al 2019** | Low | Low | Low | Low | Low | Low | Low |
| **Chauhan et al 2020** | Low | Low | Some concerns | Some concerns | Low | Low | Low |
| **Chen et al 2011** | Low | Low | Low | Low | Low | Low | Some concerns |
| **Chibarro et al 2005** | Low | Some concerns | Low | Low | Low | Low | Some concerns |
| **Cordes et al 2010** | Low | Low | Low | Low | Low | Low | Low |
| **De Jesus et al 2011** | Low | Low | Low | Low | Low | Low | Some concerns |
| **Dlabac-de Lange et al 2014** | Low | Low | Low | Low | Low | Low | Low |
| **Dollfus et al 2018** | Low | Low | Low | Low | Some concerns | Low | Some concerns |
| **Duan et al 2013** | Some concerns | Some concerns | Some concerns | Some concerns | Some concerns | Some concerns | Some concerns |
| **Fitzgerald et al 2008** | Low | Low | Some concerns | Low | Low | Low | Some concerns |
| **Gan et al 2014a** | Low | Some concerns | Some concerns | Low | Some concerns | Low | Some concerns |
| **Gan et al 2014b** | Low | Some concerns | Some concerns | Low | Some concerns | Low | Some concerns |
| **Gan et al 2015** | Low | Some concerns | Some concerns | Low | Low | Low | Some concerns |
| **Gan et al 2021** | Low | Low | Low | Low | Some concerns | Low | Some concerns |
| **Garg et al 2016** | Low | Some concerns | Some concerns | Low | Low | Low | Low |
| **Goyal et al 2007** | Low | Some concerns | Low | Low | Low | Low | Some concerns |
| **Güleken et al 2020** | Low | Some concerns | Low | Low | Some concerns | Low | Some concerns |
| **Hajak et al 2004** | Low | Low | Low | Low | Some concerns | Low | Low |
| **Holi et al 2004** | Low | Low | Low | Low | Some concerns | Low | Some concerns |
| **Huang et al 2016** | Low | Low | Low | Low | Low | Low | Low |
| **Jin et al 2023** | Low | Some concerns | Some concerns | Low | Low | Some concerns | Low |
| **Klein et al 1999** | Low | Low | Low | Some concerns | Low | Low | Low |
| **Kos et al 2024** | Low | Some concerns | Some concerns | Low | Some concerns | Some concerns | Low |
| **Kumar 2020** | Low | Low | Low | Low | Low | Low | Low |
| **Li et al 2016** | Low | Low | Low | Low | Low | Low | Low |
| **Lisoni et al 2022** | Low | Low | Low | Some concerns | Low | Low | Low |
| **Liu et al 2008** | Some concerns | Some concerns | Some concerns | Low | Some concerns | Some concerns | Low |
| **Ma et al 2016** | Low | Some concerns | Some concerns | Low | Low | Some concerns | Low |
| **Mogg et al 2007** | Low | Some concerns | Low | Low | Low | Low | Low |
| **Novak et al 2006** | Some concerns | High | High | Low | Low | Low | High |
| **Paillère et al 2016** | Low | Low | Low | Low | Low | Low | Low |
| **Palm et al 2016** | Low | Low | Low | Low | Low | Low | Low |
| **Pan et al 2021** | Some concerns | Low | Low | Low | Low | Low | Some concerns |
| **Prikryl et al 2007** | Low | Low | Low | Low | Some concerns | Some concerns | Some concerns |
| **Prikryl et al 2012** | Some concerns | High | High | Low | High | Some concerns | Low |
| **Prikryl et al 2013** | Some concerns | High | High | Low | Some concerns | Some concerns | Low |
| **Prikryl et al 2014** | Some concerns | High | High | Low | Some concerns | Some concerns | Some concerns |
| **Quan et al 2015** | Low | Low | Low | Low | Low | Low | Low |
| **Quan et al 2015** | Low | Low | Low | Low | Low | Low | Low |
| **Rabany et al 2014** | Some concerns | Some concerns | Low | Low | Low | Low | Low |
| **Ren et al 2011** | Some concerns | Some concerns | Some concerns | Some concerns | Some concerns | Some concerns | Some concerns |
| **Rosa et al 2007** | Some concerns | Low | Low | Low | Low | Low | Low |
| **Saba et al 2006** | Some concerns | Some concerns | Low | Low | Some concerns | Some concerns | Some concerns |
| **Schneider et al 2008a** | Low | Some concerns | Low | Low | Low | Low | Low |
| **Schneider et al 2008b** | Low | Some concerns | Low | Low | Low | Low | Some concerns |
| **Singh et al 2020** | Low | Low | Low | Low | Low | Low | Low |
| **Tikka et al 2017** | Low | Low | Low | Some concerns | Low | Low | Low |
| **Wang et al 2015** | Low | Low | Low | Low | Some concerns | Low | Some concerns |
| **Wang et al 2020** | Some concerns | Some concerns | Some concerns | Low | High | Low | Low |
| **Wen et al 2021** | Low | Low | Low | Low | Low | Low | Low |
| **Wen-Xiang et al 2012** | Low | Some concerns | Low | Low | Low | Low | Some concerns |
| **Wobrock et al 2015** | Low | Low | Low | Low | Low | Low | Low |
| **Xiu et al 2020a** | Low | Some concerns | Low | Low | Some concerns | Low | Some concerns |
| **Xiu et al 2020b** | Low | Some concerns | Low | Low | Some concerns | Low | Some concerns |
| **Xu et al 2006** | Low | Some concerns | Low | Low | Low | Low | Low |
| **Xu et al 2015** | Low | Some concerns | Low | Low | Low | Low | Low |
| **Zhang et al 2010** | Low | Some concerns | Low | Low | Low | Low | Low |
| **Zhang et al 2015** | Low | Some concerns | Some concerns | Some concerns | Low | Low | Some concerns |
| **Zhao et al 2014a** | Low | Low | Low | Some concerns | Some concerns | Some concerns | Some concerns |
| **Zhao et al 2014b** | Low | Low | Low | Some concerns | Some concerns | Some concerns | Some concerns |
| **Zhao et al 2014c** | Low | Low | Low | Some concerns | Some concerns | Some concerns | Some concerns |
| **Zheng et al 2012a** | Low | Some concerns | Low | Low | Low | Low | Low |
| **Zheng et al 2012b** | Low | Some concerns | Low | Low | Low | Low | Low |
| **Zheng et al 2012c** | Low | Some concerns | Low | Low | Low | Low | Low |
| **Zhu et al 2021** | Low | Low | Low | Low | Low | Low | Some concerns |
| **Zhuo et al 2019** | Low | Some concerns | Low | Low | Some concerns | Low | Low |
| **COGNITIVE SYMPTOMS: Study** | **Random sequence generation (selection bias)** | **Allocation concealment (selection bias)** | **Blinding of participants and personnel (performance bias)** | **Blinding of outcome assessment (detection bias)** | **Incomplete outcome data (attrition bias)** | **Selective reporting (reporting bias)** | **Other bias** |
| **Barr et al 2013** | Low | Low | Low | Low | Some concerns | Low | Low |
| **Francis et al 2018** | Low | Low | Low | Low | Low | Low | Low |
| **Guan et al 2020** | Low | Low | Low | Low | Some concerns | Low | Low |
| **Hasan et al 2015** | Low | Low | Low | Low | Low | Low | Low |
| **Jeon et al 2018** | Low | Low | Low | Low | Low | Low | Low |
| **Rabany et al 2014** | Some concerns | Some concerns | Low | Low | Low | Low | Low |
| **Voineskos et al 2021** | Low | Low | Low | Low | Low | Some concerns | Low |
| **Wang et al 2022** | Low | Some concerns | Low | Low | Some concerns | Low | Some concerns |
| **Wen et al 2021** | Low | Low | Low | Low | Low | Low | Low |
| **Wölwer et al 2014** | Low | Some concerns | Low | Low | Low | Low | Some concerns |
| **Xiu et al 2020a** | Low | Low | Low | Low | Low | Some concerns | Low |
| **Xiu et al 2020b** | Low | Low | Low | Low | Low | Some concerns | Low |
| **Xue et al 2023** | Low | Some concerns | Low | Low | Some concerns | Low | Some concerns |
| **Ye et al 2024a** | Low | Some concerns | Low | Low | Some concerns | Low | Some concerns |
| **Ye et al 2024b** | Low | Some concerns | Low | Low | Some concerns | Low | Some concerns |
| **Zhou et al al 2024** | Low | Some concerns | Some concerns | Low | Low | Low | Some concerns |
| **Zhuo et al 2019a** | Some concerns | Low | Low | Low | Some concerns | Low | Some concerns |
| **POSITIVE SYMPTOMS: Study** | **Random sequence generation (selection bias)** | **Allocation concealment (selection bias)** | **Blinding of participants and personnel (performance bias)** | **Blinding of outcome assessment (detection bias)** | **Incomplete outcome data (attrition bias)** | **Selective reporting (reporting bias)** | **Other bias** |
| **Brunelin et al 2012** | Low | Low | Low | Low | Low | Low | Low |
| **Chang et al 2018** | Low | Low | Low | Low | Low | Low | Low |
| **Chibarro et al 2005** | Low | Low | Low | Low | Low | Low | Low |
| **Fitzgerald et al 2005** | Low | Low | Low | Low | Low | Low | Low |
| **Fitzgerald et al 2014** | Low | Low | Low | Low | Low | Low | Low |
| **Goyal et al 2007** | Low | Low | Low | Low | Low | Low | Low |
| **Hoffman et al 2005** | Low | Low | Low | Low | Low | Some concerns | Low |
| **Hoffman et al 2013** | Low | Low | Low | Low | Some concerns | Some concerns | Low |
| **Holi et al 2004** | Low | Low | Low | Low | Low | Low | Low |
| **Hua et al 2024** | Low | Low | Low | Low | Some concerns | Low | Some concerns |
| **Koops et al 2016** | Low | Low | Low | Low | Low | Low | Low |
| **Koops et al 2018** | Low | Low | Low | Low | Low | Low | Low |
| **Lee et al 2004** | Low | Some concerns | Low | Low | Low | Low | Some concerns |
| **Macintosh et al 2004** | Some concerns | Some concerns | Some concerns | Some concerns | Low | High | High |
| **Martinot et al 2016** | Low | Low | Low | Low | Low | Low | Low |
| **Ray et al 2015** | Low | Low | Low | Low | Low | Low | Low |
| **Rosenberg 2012** | Low | Low | Low | Low | Low | Low | Low |
| **Saba et al 2006** | Low | Low | Low | Low | Low | Low | Low |
| **Slotema et al 2011** | Low | Some concerns | Some concerns | Low | Low | Low | Low |
| **Tikka et al 2018** | Low | Low | Low | Some concerns | Low | Low | Low |
| **Tyagi et al 2022** | Low | Low | Low | Some concerns | Low | Low | Low |
| **Vercammen et al 2009** | Low | Low | Low | Low | Low | Low | Low |
| **Yuanjun et al 2024** | Some concerns | Some concerns | Some concerns | Low | Some concerns | Some concerns | Some concerns |
| **Zhu et al 2021** | Low | Low | Low | Low | Low | Low | Low |

Table S5. Breakdown of sample sizes across all included studies

Studies with < 50 participants (total = 68 studies)

| **Study** | **n (TMS)** | **n (sham)** | **n (total)** |
| --- | --- | --- | --- |
| **NEGATIVE SYMPTOMS** | | | |
| **Bais et al 2014** | 31 | 16 | 47 |
| **Barr et al 2012** | 13 | 12 | 25 |
| **Bation et al 2021** | 12 | 10 | 22 |
| **Brady et al 2019** | 8 | 3 | 11 |
| **Chauhan et al 2020** | 19 | 17 | 36 |
| **Chen et al 2011** | 23 | 19 | 42 |
| **Chibarro et al 2005** | 8 | 8 | 16 |
| **Cordes et al 2010** | 12 | 13 | 25 |
| **De Jesus et al 2011** | 8 | 9 | 17 |
| **Dlabac-de Lange et al 2014** | 16 | 16 | 32 |
| **Duan et al 2013** | 21 | 20 | 41 |
| **Fitzgerald et al 2008** | 12 | 8 | 20 |
| **Gan et al 2014a** | 20 | 21 | 41 |
| **Gan et al 2021** | 17 | 16 | 33 |
| **Garg et al 2016** | 20 | 20 | 40 |
| **Goyal et al 2007** | 5 | 5 | 10 |
| **Guan et al 2020** | 21 | 20 | 41 |
| **Güleken et al 2020** | 11 | 10 | 21 |
| **Hajak et al 2004** | 10 | 10 | 20 |
| **Holi et al 2004** | 11 | 11 | 22 |
| **Huang et al 2016** | 19 | 18 | 37 |
| **Klein et al 1999** | 16 | 15 | 31 |
| **Kos et al 2024a** | 32 | 16 | 48 |
| **Kos et al 2024b** | 17 | 17 | 34 |
| **Li et al 2016** | 25 | 22 | 47 |
| **Liu et al 2008** | 13 | 12 | 25 |
| **Mogg et al 2007** | 8 | 9 | 17 |
| **Novak et al 2006** | 8 | 8 | 16 |
| **Paillère et al 2016** | 15 | 12 | 27 |
| **Pan et al 2021** | 16 | 20 | 36 |
| **Prikryl et al 2007** | 11 | 11 | 22 |
| **Prikryl et al 2012** | 19 | 11 | 30 |
| **Prikryl et al 2013** | 23 | 17 | 40 |
| **Prikryl et al 2014** | 18 | 17 | 35 |
| **Rabany et al 2014** | 20 | 10 | 30 |
| **Ren et al 2011** | 12 | 11 | 23 |
| **Rosa et al 2007** | 6 | 5 | 11 |
| **Rosenberg et al 2012** | 5 | 5 | 10 |
| **Saba et al 2006** | 8 | 8 | 16 |
| **Schneider et al 2008a** | 17 | 17 | 34 |
| **Schneider et al 2008b** | 17 | 17 | 34 |
| **Singh et al 2020** | 15 | 15 | 30 |
| **Tikka et al 2017** | 8 | 7 | 15 |
| **Zhang et al 2010** | 15 | 12 | 27 |
| **Zhao et al 2014a** | 24 | 22 | 46 |
| **Zhao et al 2014b** | 23 | 22 | 45 |
| **Zhao et al 2014c** | 24 | 22 | 46 |
| **Zheng et al 2012a** | 19 | 17 | 36 |
| **Zheng et al 2012b** | 19 | 17 | 36 |
| **Zheng et al 2012c** | 18 | 17 | 35 |
| **COGNITIVE SYMPTOMS** | | | |
| **Barr et al 2013** | 13 | 14 | 27 |
| **Francis et al 2018** | 19 | 10 | 29 |
| **Guan et al 2020** | 21 | 20 | 41 |
| **Rabany et al 2014** | 20 | 10 | 30 |
| **Wölwer et al 2014** | 18 | 14 | 32 |
| **POSITIVE SYMPTOMS** | | | |
| **Chibarro et al 2005** | 8 | 8 | 16 |
| **Fitzgerald et al 2005** | 17 | 16 | 33 |
| **Goyal et al 2007** | 5 | 5 | 10 |
| **Holi et al 2004** | 11 | 11 | 22 |
| **Lee et al 2004** | 13 | 14 | 27 |
| **Macintosh et al 2004** | 8 | 8 | 16 |
| **Martinot et al 2016** | 15 | 12 | 27 |
| **Ray et al 2015** | 20 | 20 | 40 |
| **Rosenberg 2012** | 5 | 5 | 10 |
| **Saba et al 2006** | 8 | 8 | 16 |
| **Slotema et al 2011** | 22 | 20 | 42 |
| **Tikka et al 2018** | 8 | 7 | 15 |
| **Vercammen et al 2009** | 12 | 12 | 24 |

Studies with >= 50 participants (total = 42 studies)

| **Study** | **n (TMS)** | **n (sham)** | **n (total)** |
| --- | --- | --- | --- |
| **NEGATIVE SYMPTOMS** | | | |
| **Bai et al 2015** | 36 | 35 | 71 |
| **Basavaraju et al 2021** | 30 | 30 | 60 |
| **Bodén et al 2021** | 28 | 28 | 56 |
| **Dollfus et al 2018** | 26 | 33 | 59 |
| **Gan et al 2014b** | 38 | 37 | 75 |
| **Gan et al 2015** | 32 | 35 | 67 |
| **Kumar 2020** | 50 | 50 | 100 |
| **Ma et al 2016** | 60 | 58 | 118 |
| **Quan et al 2015** | 78 | 39 | 117 |
| **Quan et al 2015** | 78 | 39 | 117 |
| **Wang et al 2015** | 41 | 42 | 83 |
| **Wang et al 2020** | 25 | 25 | 50 |
| **Wen et al 2021** | 26 | 26 | 52 |
| **Wen-Xiang et al 2012** | 76 | 31 | 107 |
| **Wobrock et al 2015** | 76 | 81 | 157 |
| **Xiu et al 2020a** | 32 | 30 | 62 |
| **Xiu et al 2020b** | 35 | 30 | 65 |
| **Xu et al 2006** | 40 | 23 | 63 |
| **Xu et al 2015** | 60 | 30 | 90 |
| **Yin et al 2023** | 34 | 32 | 66 |
| **Zhang et al 2015** | 35 | 34 | 69 |
| **Zhu et al 2021** | 32 | 32 | 64 |
| **Zhuo et al 2019** | 33 | 27 | 60 |
| **COGNITIVE SYMPTOMS** | | | |
| **Hasan et al 2015** | 77 | 79 | 156 |
| **Voineskos et al 2021** | 41 | 40 | 81 |
| **Wang et al 2022** | 33 | 26 | 59 |
| **Wen et al 2021** | 26 | 26 | 52 |
| **Xiu et al 2020a** | 40 | 40 | 80 |
| **Xiu et al 2020b** | 40 | 40 | 80 |
| **Xue et al 2023** | 26 | 24 | 50 |
| **Ye et al 2024a** | 28 | 28 | 56 |
| **Ye et al 2024b** | 28 | 28 | 56 |
| **Zhou et al 2024** | 59 | 69 | 128 |
| **Zhuo et al 2019a** | 33 | 27 | 60 |
| **POSITIVE SYMPTOMS** | | | |
| **Hoffman et al 2005** | 27 | 23 | 50 |
| **Hoffman et al 2013** | 55 | 28 | 83 |
| **Hua et al 2024** | 32 | 30 | 62 |
| **Koops et al 2016** | 32 | 32 | 64 |
| **Tyagi et al 2022** | 30 | 29 | 59 |
| **Yuanjun et al 2024** | 30 | 25 | 55 |
| **Zhu et al 2021** | 32 | 32 | 64 |

Table S6. Pooled Standardized Mean Differences (SMD) Across Symptom Domains Using Random Effects, Bootstrapping, and Permutation Testing

| **Domains** | **Random Effects (SMD, 95% CI)** | **Bootstrap Mean (95% CI)** | **Permutation (SMD, p-value)** |
| --- | --- | --- | --- |
| **Main Groups:** | | | |
| All Symptoms | 0.44 [0.30, 0.57] | 0.43 [0.29, 0.57] | 0.43, p<0.00001 |
| Negative Symptoms | 0.48 [0.31, 0.65] | 0.47 [0.30, 0.65] | 0.47, p<0.00001 |
| Cognitive Symptoms | 0.50 [0.30, 0.71] | 0.51 [0.32, 0.74] | 0.50, p=0.0002 |
| Positive Symptoms | 0.24 [-0.17, 0.64] | 0.22 [-0.21, 0.55] | 0.22, p=0.316 |
| **Negative symptoms subgroups:** | | | |
| High frequency | 0.52 [0.34, 0.70] | 0.50 [0.34, 0.70] | 0.51, p<0.00001 |
| Low frequency | 0.06 [-0.31, 0.44] | 0.07 [-0.21, 0.50] | 0.06, p=0.751 |
| Left PFC - High frequency | 0.62 [0.41, 0.83] | 0.61 [0.40, 0.86] | 0.62, p<0.00001 |
| Left PFC - iTBS | 1.01 [0.44, 1.58] | 1.00 [0.52, 1.56] | 0.99, p=0.0134 |
| Left PFC - 20Hz | 0.56 [0.11, 1.00] | 0.59 [0.20, 1.16] | 0.57, p=0.0312 |
| Left PFC - 10Hz | 0.52 [0.24, 0.80] | 0.52 [0.26, 0.82] | 0.52, p=0.0008 |
| Cerebellum | 0.13 [-0.64, 0.90] | 0.02 [-0.49, 0.56] | 0.00, p=1 |
| Left TPC | 0.08 [-0.27, 0.42] | 0.09 [-0.17, 0.45] | 0.07, p=0.679 |
| **Cognitive symptoms subgroups:** | | | |
| Left PFC | 0.43 [0.26, 0.60] | 0.43 [0.28, 0.60] | 0.43, p=0.0002 |
| **Positive symptoms subgroups:** | | | |
| High frequency | 0.23 [-0.05, 0.52] | 0.26 [0.05, 0.54] | 0.23, p=0.125 |
| Low frequency | 0.18 [-0.42, 0.78] | 0.19 [-0.39, 0.70] | 0.16, p=0.596 |
| Left TPC | 0.44 [0.10, 0.78] | 0.44 [0.16, 0.77] | 0.44, p=0.015 |
| Left TPC: Low frequency | 0.49 [0.12, 0.86] | 0.50 [0.21, 0.82] | 0.49, p=0.015 |
| *Abbreviations: PFC = prefrontal cortex, TPC = temporoparietal cortex, iTBS = intermittent theta burst stimulation* | | | |

**References:**

[1] Wang J, Zhou Y, Gan H, Pang J, Li H, Wang J, et al. Efficacy Towards Negative Symptoms and Safety of Repetitive Transcranial Magnetic Stimulation Treatment for Patients with Schizophrenia: A Systematic Review. Shanghai Arch Psychiatry. 2017 Apr 25;29(2):61.

[2] Lorentzen R, Nguyen TD, McGirr A, Hieronymus F, Østergaard SD. The efficacy of transcranial magnetic stimulation (TMS) for negative symptoms in schizophrenia: a systematic review and meta-analysis. Schizophrenia. 2022 Apr 9;8(1):35.
